# A Systematic Literature Review of Coordinated Care in Cardiovascular-Kidney-Metabolic Conditions

**DOI:** 10.1016/j.mayocpiqo.2025.100671

**Published:** 2025-11-12

**Authors:** O. Kenrik Duru, Radica Alicic, Muthiah Vaduganathan, Wendy L. St. Peter, Glenda V. Roberts, Janani Rangaswami, Susanne B. Nicholas, Joshua J. Neumiller, Roy O. Mathew, Patrick Gee, Katherine R. Tuttle

**Affiliations:** a Division of General Internal Medicine and Health Services Research, David Geffen School of Medicine, Los Angeles, CA; b Department of Medicine, Division of Nephrology, David Geffen School of Medicine, Los Angeles, CA; c Providence Medical Research Center, Providence Inland Northwest Health, Spokane, WA; d Department of Medicine, University of Washington School of Medicine, Seattle, WA; e Nephrology Division and Kidney Research Institute, University of Washington School of Medicine, Seattle, WA; f Brigham and Women’s Hospital Heart & Vascular Center, Harvard Medical School, Boston, MA; g Department of Pharmaceutical Care and Health Systems, College of Pharmacy, University of Minnesota, Minneapolis, MN; h Barbara T. Murphy Division of Nephrology, Icahn School of Medicine at Mount Sinai, New York City, NY; i Division of Nephrology, Washington DC VA Medical Center, Washington, DC; j Department of Pharmacotherapy, College of Pharmacy and Pharmaceutical Sciences, Washington State University, Spokane, WA; k Loma Linda VA Health Care System, Loma Linda, CA; l iAdvocate Inc, P Gee Consulting, LLC, Greater Richmond Region, VA

## Abstract

**Objective:**

To assess coordinated cardiovascular-kidney-metabolic (CKM) care programs, including program types, components, and outcomes.

**Patients and Methods:**

We searched Embase and Medline for studies from January 1, 2015 through March 9, 2023, and congress abstracts from January 1, 2021 through March 9, 2023. For inclusion, patients were required to have ≥2 CKM conditions and the coordinated care program assessed the effectiveness of either treatment, monitoring, or risk reduction of all 3 conditions. Two reviewers extracted and assessed the data for accuracy. Randomized controlled trials were assessed for potential bias in the design, conduct, and reporting of clinical trials risk of bias using the Cochrane risk of bias tool, version 2. Observational studies were assessed using the Newcastle-Ottawa Scale.

**Results:**

A total of 22 international studies met our inclusion criteria; interventions included patient visits to multidisciplinary team (MDT) care clinics (n=9), pharmacist integration (n=5), patient engagement and education (n=6), or MDT/multispecialty team meetings (n=2). The sample size of studies ranged from 14 to 9601. Overall, results showed greater patient satisfaction and fewer health-related problems with coordinated care programs versus usual care, with increased attendance rates and decreased health care costs for virtual consultations, and further reductions for programs integrating telehealth.

**Conclusion:**

Coordinated care for patients with CKM conditions may improve clinical outcomes and reduce healthcare costs. Future research is needed to develop programs with standard reporting, to assess overall effectiveness, and to identify best practices for implementing coordinated care programs. Limitations included heterogeneity in the interventions’ design, delivery, CKM population, and outcomes assessed.

**Trial Registration:**

PROSPERO Identifier: CRD42023409731

## Graphical abstract

**Figure:**
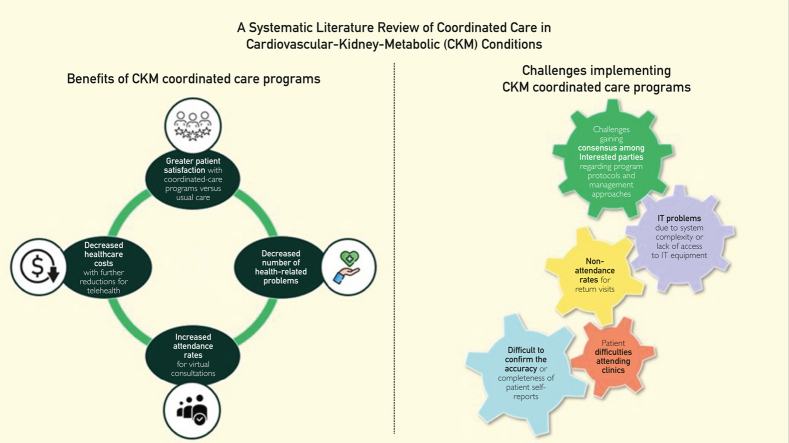

**Article Highlights**

• Cardiovascular-kidney-metabolic (CKM) syndrome is a group of interrelated health disorders associated with increased risk of morbidity and mortality. Recent guidelines stipulate the need for coordinated CKM care.
• Coordinated CKM care programs report greater patient satisfaction, fewer health-related problems, increased attendance rates, and decreased health care costs for virtual consultations, with further costs reductions for programs integrating telehealth.
• Future research is needed to develop coordinated care programs with standard reporting, assessment of effectiveness with contemporaneous controls and to identify components that are most beneficial along with implementation methods.

In 2023, the American Heart Association (AHA) provided guidance for the definitions, staging, prediction strategies, and algorithms for the prevention and treatment of cardiovascular-kidney-metabolic (CKM) syndrome, a group of interrelated health disorders comprising obesity, metabolic syndrome, type 2 diabetes (T2D), hypertension, dyslipidemia, chronic kidney disease (CKD), and cardiovascular diseases.^1^, ^2^, ^3^ CKM syndrome is associated with increased risk of morbidity and mortality, with greater risks in patients with multiple CKM conditions.^4^ These conditions share many traditional risk factors, such as older age, male sex, smoking, and a family history along with elevated systemic inflammation, oxidative stress, and neurohumoral activation.^5^^,^^6^ In the United States, the prevalence of CKM conditions is projected to increase from 2020 to 2050, including obesity (43.1%-60.6%), T2D (16.3%-26.8%), hypertension (51.2%-61.0%), and heart failure (2.7%-3.8%).^7^ Patients with CKM syndrome are at a high-risk of multiple adverse cardiovascular and kidney events. Thus, strategies to reduce CKM risks and optimize treatment are needed.

The American Diabetes Association, the American Society of Nephrology, Kidney Disease: Improving Global Outcomes, and the AHA have emphasized the need for coordinated CKM care that is comprehensive, holistic, and patient-centered.^2^^,^^3^^,^^8^, ^9^, ^10^, ^11^ In a successful coordinated care program, patients with CKM syndrome would receive treatment from a multidisciplinary team (MDT) comprising specialists (cardiology, nephrology, and endocrinology) partnering with primary care practitioners, pharmacists, nurses, care navigators, and allied health professionals (such as nutritionists and occupational therapists). Programs may involve ongoing care via virtual or in-person interactions, regular review of health records, aligned treatment algorithms, incorporation of patient preferences and priorities, and operational viability to achieve therapeutic goals.^12^ However, types of coordinated care programs for CKM syndrome are not well characterized. Although narrative reviews have assessed models of care for patients with CKM conditions, systematic literature reviews (SLRs) conducted on this topic are lacking. This SLR assessed coordinated CKM care by describing program types, components, outcomes, effectiveness, implementation challenges, and facilitators (Graphical Abstract).

## Patients and Methods

This SLR was conducted using a standardized methodical, comprehensive, and transparent approach following guidance presented in the Preferred Reporting Items for Systematic Reviews and Meta-Analyses (PRISMA) 2020 statement.^13^ The protocol followed the PRISMA Protocol guidelines and defined all processes and methodologies used to conduct this SLR. The protocol was prospectively registered with PROSPERO (International Prospective Register of Systematic Reviews) to avoid duplication and reduce potential reporting bias (registration number: CRD42023409731). No changes were made to the protocol except that 1 author’s conflict of interest disclosure was amended upon their request, and an additional author was added.

### Inclusion Criteria

Relevant evidence was identified to meet the review objectives and prespecified population, intervention, comparator, outcome, and study design types eligibility criteria. For the purpose of this SLR, CKM conditions included cardiovascular diseases, CKD, and metabolic conditions (T2D, overweight, or obesity). Eligible studies enrolled participants with at least 2 of the 3 of these CKM conditions, and the tested intervention assessed the effectiveness of treatment, monitoring, or risk prevention of all 3 CKM conditions. English-language studies published from January 1, 2015 (to correspond with the first publications of cardiovascular outcomes trials in patients with T2D), through March 9, 2023, or congress abstracts published from January 1, 2021 through March 9, 2023, were eligible for inclusion. Detailed inclusion and exclusion criteria are reported in Table 1.

**Table 1:** Summary of Study Inclusion Criteria

	Inclusion criteria	Exclusion criteria
Population	• Participants (any age group) with or being monitored for ≥2 CKM conditions, enrolled in a treatment program for CKM^a^ or receiving care from a CKM MDT	• Not a participant in a CKM program or receiving care from a CKM MDT • Participants with <2 CKM conditions
Intervention/comparator	• Coordinated care for CKM or an MDT approach^a^	• Coordinated care/MDT that does not address all 3 CKM conditions
Outcomes	• Type, components, benefits, and effectiveness of coordinated care program • Resources required and implementation challenges	• NA
Study types	• RWE/observational studies • Phase 2-4 clinical trials • Study protocols	• Preclinical studies, pilot or phase 1 clinical studies, case reports/studies, notes, commentaries, letters, editorials, opinions, guidelines, meta-analyses, and reviews^b^
Other	• Studies published in English • Journal articles published from 2015 to March 2023^c^ • Congress abstracts published from January 2021 to March 2023	• Non-English publications or unpublished data • Journal articles published before 2015^c^ • Congress abstracts published before 2021 • Journal abstracts and congress abstracts published after March 9, 2023

CKM, cardio-kidney-metabolic; MDT, multidisciplinary team; NA, not applicable; RWE, real-world evidence.

a Coordinated care/MDT approach must have components that address all 3 CKM conditions. At least 2 of the 3 CKM conditions must be reported in the abstract to be considered for full-text review.

b Reviews were excluded but reference lists of relevant systematic reviews were screened for primary sources.

c Studies published from 2015 to correspond with cardiovascular outcome trial data.

### Searches, Screening, and Data Extraction

Systematic searches were conducted in Embase and Medline using a prespecified search strategy (Supplemental Table 1, available online at http://www.mcpiqojournal.org). One reviewer conducted screening of titles and abstracts according to inclusion and exclusion criteria, with a 20% check by a second reviewer. Agreement on included studies during title/abstract screening was high at 97%. Where there was disagreement, the record was retained to full-text screening. The same approach was taken for full-text screening. The full-text screening agreement rate was 86%, and so, the second reviewer screened 50% of the full-text reports. Discrepancies were resolved through discussion with a third, senior reviewer.

### Data Extraction and Quality Appraisal

Publication details, population characteristics, intervention details, and outcomes data from the included studies were extracted into a prespecified data extraction table. Data extraction was performed by 1 reviewer and assessed for accuracy by a second reviewer. Randomized controlled trials (RCTs) were assessed for potential bias in the design, conduct, and reporting of clinical trials using the Cochrane risk of bias tool, version 2, for randomized trials.^14^ Observational studies were assessed using the Newcastle-Ottawa Scale (NOS)^15^ for cohort studies, which appraises each study on 8 items in 3 categories: cohort selection, comparability between exposed and nonexposed cohorts, and quality of outcome reporting.^15^ Only full-text publications with results were quality assessed; study protocols and congress abstracts were not quality assessed due to limited data. Because the data collected were expected to be qualitative and very heterogeneous, no meta-analysis was planned because the data would not be suitable for any type of pooled analysis.

## Results

### Geographical Distribution of the Included Studies

In total, 1553 records were identified. A summary of studies included at each stage of the screening process and reasons for exclusion are shown in the PRISMA diagram (Figure 1). Twenty-two interventions, reported in 25 publications (19 full-text [including 2 protocols] and 6 congress abstracts) met eligibility criteria and were included in this SLR (Table 2).^16^, ^17^, ^18^, ^19^, ^20^, ^21^, ^22^, ^23^, ^24^, ^25^, ^26^, ^27^, ^28^, ^29^, ^30^, ^31^, ^32^, ^33^, ^34^, ^35^, ^36^, ^37^, ^38^, ^39^, ^40^ Interventions were conducted in the United States (7/22),^16^, ^17^, ^18^, ^19^, ^20^, ^21^, ^22^, ^23^ the United Kingdom (4/22),^24^, ^25^, ^26^, ^27^, ^28^ Australia (2/22),^29^^,^^30^ Canada (2/22),^31^^,^^32^ and 1 each in Germany,^33^ Sweden,^34^^,^^35^ Italy,^36^ Singapore,^37^ Thailand,^38^ and Hong Kong,^39^ and 1 in 8 countries in Asia (China, Hong Kong, Malaysia, Philippines, South Korea, Taiwan, Thailand, and Vietnam).^40^

**Figure:**
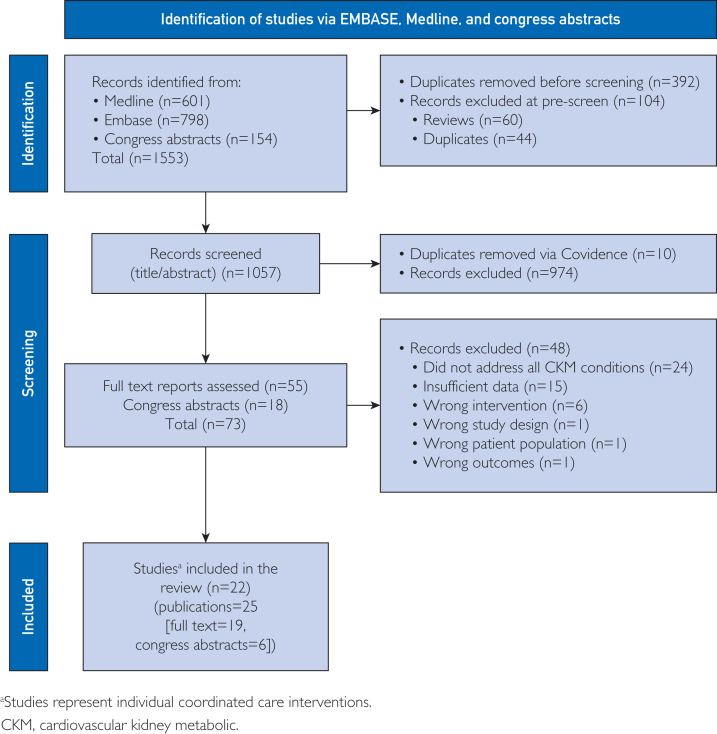
Preferred Reporting Items for Systematic Reviews and Meta-Analyses (PRISMA) flow chart.CKM, cardiovascular-kidney-metabolic.

**Table 2:** Study Characteristics, Grouped by Setting (n=22)

Reference, year (country)	Intervention/study name (NCT)	Intervention type	Target CKM population	Intervention setting	Study design (study period)/overlap with COVID-19 pandemic	Intervention details	Virtual/in-person
Outpatient specialist clinic (n=14 interventions in 15 publications)							
Al-Chalabi et al,^24^ 2022 (UK)	Metabolic-Renal-Cardiac (MRC) clinic	Patient visits	All 3 CKM conditions	MRC clinic	Single-center retrospective cohort analysis (Mar 2021-Nov 2021) During COVID-19	MRC clinic provided diabetology and cardiology input in addition to nephrology care for patients with cardiorenal syndrome	In-person
Comaschi et al,^36^ 2020 (Italy)	INSIDE study (protocol)	Patient visits	T2DM	Primary care units and specialist diabetes and cardiology centers throughout Italy	Protocol for multicenter RCT (NA) Overlap with COVID-19: NA	Integrates primary care, diabetes care, and CVD care. Includes kidney function monitoring	In-person
Chan et al,^40^ 2022 (Asia^a^)	A Joint Asia Diabetes Evaluation (JADE) RCT (NCT02176278)	Patient engagement/education	DKD	Hospital-based diabetes centers	RCT (Jun 2014-Feb 2019) Pre–COVID-19	JADE—MDT provided self-management education and outreach for patients with DKD to meet HbA1c, kidney function, and BP targets	Web-based platform, telephone outreach, in-person assessments
Dubrofsky et al,^31^ 2022 (Canada)	C.a.R.E. Clinic	Patient visits	All 3 CKM conditions	C.a.R.E. clinic	Single-center retrospective cohort (Jul 2014-Feb 2020) Pre–COVID-19	C.a.R.E. clinic allows patients to see multiple providers on same day Cardiologist, nephrologist, and endocrinologist develop care plan for patients with T2DM and CVD and/or CKD	In-person
Jegatheesan et al,^29^ 2022 (Australia)	LANDMARK III (multidisciplinary lifestyle intervention)	Patient engagement/education Patient visits	CKD	NR (outpatient specialty clinic assumed)	RCT (NR) Overlap with COVID-19: cannot determine	LANDMARK III (multidisciplinary lifestyle intervention) for managing cardiac risk factors in patients with CKD through lifestyle and behavioral modifications, with an MDT/MST including a nurse, exercise physiologist, dietitian, psychologist, diabetes educator, and social worker	In-person
Keel et al,^34^ 2020 (Sweden)	CareHND center (NCT03362983)	Patient visits	All 3 CKM conditions	HND Center	Mixed-method approach to cost analysis (2017) Pre–COVID-19	CareHND: A multidisciplinary clinic focused on providing care to patients with all 3 CKM conditions at a single location	In-person
Rafiq et al,^35^ 2019 (Sweden)	CareHND (NCT03362983)	Patient visits	All 3 CKM conditions	HND center	RCT (Jan 2008-Apr 2018) Pre–COVID-19	CareHND: A multidisciplinary clinic focused on providing care to patients with all 3 CKM conditions at a single location	In-person
Li and Radhakrishnan,^19^ 2021 (USA)	Pharmacist–physician collaborative care	Pharmacist integration	CKD	NR (outpatient specialty clinic assumed)	Single-center observational (NR) Overlap with COVID-19: cannot determine	Nephrologist–pharmacist collaboration to incorporate pharmacist-driven medication management for HTN and T2DM in patients with CKD, using technology^b^ to improve outcomes	Virtual
Lim et al,^39^ 2020 (Hong Kong)	Technology-assisted integrated care	Patient engagement/education	T2DM	Outpatient clinics and diabetes centers	Retrospective cohort analysis (enrolled: 2007-2015; follow-up through June 30, 2017) Pre–COVID-19	Technology-assisted integrated care using JADE–MDT providing self-management education and outreach for patients with T2DM to meet HbA1c, kidney function, and BP targets	Web-based platform, telephone outreach, and in-person assessments
Narain et al,^27^ 2022 (UK)	Cardiometabolic Clinic (CMC)	Patient visits	Any 2 of 3	CMC	Single-center retrospective cohort (Sep 2020-Mar 2022) During COVID-19	CMC: MDT review of HF/T2DM patients records to facilitate medication optimization and lifestyle interventions. MDT recommends next steps, for example, referrals to other specialists or initiating/changing/stopping medication	In-person planned, delivered virtually due to COVID-19
Neeland et al,^21^ 2022 (USA)	CINEMA program	Patient visits	T2DM	Outpatient clinic	Prospective cohort study (interim analysis) (May 2020-Aug 2021)	CINEMA program: Care team visits (in-person or virtually) the patient with T2DM at risk of CVD and/or CKD for initial assessment, followed by continued support via telephone or virtual meetings	In-person and virtual
Schutze et al,^33^ 2021 (Germany)	NR (adding clinical pharmacist to the therapeutic team)	Pharmacist integration, GP training/education	CKD	Nephrology outpatient department and department of pharmacy	Single-center prospective controlled interventional trial (June 2015-May 2019) Pre–COVID-19	Nephrologist–pharmacist collaboration to devise and propose medication plan to GP for patients with CKD and HTN and/or T2DM. Pharmacist follows up with patient after 3 mo	In-person
Tan et al,^37^ 2019 (Singapore)	IDEALS program (NCT03413215)	Patient engagement/education	DKD	Outpatient clinic	Protocol (NA) Overlap with COVID-19: NA	IDEALS program (protocol) MDT will assess and counsel patients with T2DM in self-management, provide self-management resources,^c^ and adjust medications as needed to meet BP, kidney function, and HbA1c targets	In-person with telephone follow-up and remote monitoring
Triantafylidis et al,^22^ 2021 (USA)	NR (embedding clinical pharmacist within interprofessional nephrology clinic)	Pharmacist integration	CKD	Outpatient nephrology clinic	Single-center prospective cohort (NR) Overlap with COVID-19: cannot determine	Pharmacist integration within nephrology clinic for initiation and monitoring of empagliflozin in patients with DKD. Pharmacist also provides patient education and telephone follow-up and collaborates with nephrologist to adjust T2DM, HTN, and diuretic medications as needed	In-person
Wongprasert,^38^ 2022 (Thailand)	MDT care + patient empowerment (NCT: NR)	Patient engagement/education	DKD	NR (outpatient clinic)	RCT (2 y) During COVID-19	MDT care + patient empowerment program: patients with T2DM received MDT care, education, and self-management resources^b^ to manage their own care and reduce CVD and CKD risk (program appears similar to IDEALS protocol^37^)	In-person
Primary care practices (n=3 interventions in 4 publications)							
Anderegg et al,^16^ 2018 (USA)	CAPTION trial (NCT00935077)	Pharmacist integration	HTN	Primary care practices	Post hoc analysis of RCT (Mar 2010-Jun 2013) Pre–COVID-19	CAPTION RCT: program to improve BP in hypertensive patients with T2DM and/or CKD	In-person
Dixon et al,^18^ 2021 (USA)	CAPTION trial (NCT00935077)	Pharmacist integration	HTN	Primary care practices	Post hoc analysis of RCT (NR [2010-2014]) Pre–COVID-19	CAPTION RCT: Program to improve BP in hypertensive patients with T2DM and/or CKD	In-person
Lear et al,^32^ 2021 (Canada)	iCDM (NCT01342263)	Patient engagement/education Behavioral change	Any 2 of 3	Primary care clinics	RCT (Oct 2011-Mar 2015) Pre–COVID-19	iCDM: Patients with ≥2 of T2DM, HF, IHD, CKD, or COPD access iCDM website to complete symptoms report. Alerts generated and patients provided support or hospital referral if required. Patients also provided access to public forum, their care plan, and external educational resources	Virtual
Vu et al,^23^ 2023 (USA)	Positive Kidney Health	Pharmacist integration	CKD	Primary care clinics	Prospective cohort study (Feb 2021-Oct 2021) During COVID-19	Positive Kidney Health: Pharmacist integration in primary care to educate patients with CKD to self-manage risk factors including T2DM and HTN. Pharmacists met with patients in-person or by video consultation and assessed medications/recommended changes	In-person or virtual
Primary/telemedicine with MDT (n=3 interventions in 3 publications)							
Burke et al,^17^ 2023 (USA)	NR (telemedicine between primary care site and multispecialty distance site)	Patient visits	Any 2 of 3	Primary care + virtual MDT	Prospective cohort (Mar 2017-Mar 2020) Pre–COVID-19	Telemedicine between primary care site and multispecialty distance site for patients in underserved urban areas	Virtual and in-person
Katz et al,^30^ 2018 (Australia)	iConnect CKD	Patient visits	CKD	iConnect Care	Single-center retrospective observational and RCT (Jul 2013-Sep 2015) Pre–COVID-19	iConnect CKD: a web-based CKD, HTN, and T2DM integrated care program. GPs virtually consult with nephrologist for patients with CKD including those with T2DM or CVD. Specialists review patients’ data in the web-based system, refer to additional specialists if needed, and develop a report for the GPs to follow-up with patients	Virtual
Lu et al,^20^ 2021 (USA)	V-IMPACT	Patient visits	T2DM	Outpatient	Retrospective observational (Jan 2018-Dec 2019) Pre–COVID-19	V-IMPACT: a hub-and-spoke model, where hub team of specialists provide video consultations to patients with T2DM at risk of HTN and/or CKD at the primary care clinic	Virtual
MDT meetings (n=2 interventions in 3 publications)							
Essa et al,^25^ 2021 (UK)	NR (HF multispecialty MDT virtual meetings)	MDT meetings (virtual)	CVD (HF)	Outpatient and hospital: HF multispecialty clinic	Single-center retrospective observational (Jan 2020-Dec 2020) During COVID-19	Virtual MST/MDT (including cardiologist, nephrologist, and endocrinologist) consultation meetings to optimize care for patients with HF	Virtual
Essa et al,^26^ 2022 (UK)	NR (HF multispecialty MDT virtual meetings)	MDT meetings (virtual)	CVD (HF)	Outpatient and hospital: HF multispecialty clinic	Single-center retrospective observational (Jan 2020-Jun 2021) During COVID-19	Virtual MST/MDT (including cardiologist, nephrologist, endocrinologist) consultation meetings to optimize care for patients with HF	Virtual
Saied et al,^28^ 2022 (UK)	NR (HF multispecialty MDT)	MDT/MST meetings	CVD (HF)	Multispecialty HF MDT	Single-center retrospective cohort (Jan 2020-Dec 2020) During COVID-19	MST/MDT including HF specialist, nephrologist, diabetes specialist, pharmacist, nurses: goal is to reduce polypharmacy burden and adverse consequences in patients with HF	In-person

Publications highlighted in similar colors represent the same interventions.

BP, blood pressure; CAPTION, Collaboration Among Pharmacists and Physicians to Improve Outcomes Now; C.a.R.E., Cardiac and Renal Endocrine; CareHND, Care Heart Nephrology Diabetes; CINEMA (program), Center for Integrated and Novel Approaches in Vascular-Metabolic Disease; CKD, chronic kidney disease; CKM, cardiovascular kidney metabolic; CMC, cardiometabolic clinic; COPD, chronic obstructive pulmonary disease; CVD, cardiovascular disease; DKD, diabetic kidney disease; GP, general practitioner; HbA1c, glycated hemoglobin; HF, heart failure; HND, Heart Nephrology Diabetes (center); HTN, hypertension; iCDM, Internet-based Platform for Chronic Diseases Management; IDEALS, Integrated Diabetes Education, Awareness and Lifestyle modification in Singapore; IHD, ischemic heart disease; INSIDE (study), INtegration of care for reaching targetS In Diabetic patiEnts; JADE, Joint Asia Diabetes Evaluation; MDT, multidisciplinary team; MRC, metabolic-renal-cardiac; MST, multispecialty team; NA, not applicable; NCT, national clinical trial; NR, not reported; RCT, randomized controlled trial; T2DM, type 2 diabetes mellitus; UK, United Kingdom; USA, United States of America; V-IMPACT, Virtual Integrated Multisite Patient Aligned Care Team.

a 8 countries or regions in Asia: China, Hong Kong, Malaysia, Philippines, South Korea, Taiwan, Thailand, Vietnam.

b Patients received a blood pressure monitor kit connected to tablet device for home measurements; data were transmitted to electronic medical record for team to review regularly.

c Self-management resources include glucometer and blood pressure monitor in addition to education from MDT.

### Study Design

Eight of the 22 studies, reported in 10 publications, were RCTs (5 empirical research RCTs,^29^^,^^32^^,^^35^^,^^38^^,^^40^ 2 were post hoc analyses,^16^^,^^18^ 2 were protocols,^36^^,^^37^ and 1 was a cost analysis^34^), 13 were observational (5 prospective^17^^,^^21^^,^^23^^,^^37^^,^^38^ and 9 retrospective^19^^,^^20^^,^^24^, ^25^, ^26^, ^27^, ^28^^,^^31^^,^^39^) studies, and 1 was both observational and an RCT.^30^ The studies by Anderegg et al^16^ and Dixon et al,^18^ both post hoc analyses, were linked publications of the CAPTION trial, whereas those by Rafiq et al^35^ (care utilization) and Keel et al^34^ (cost analysis) were linked publications of the CareHND trial. The studies by Essa et al^25^^,^^26^ in 2021 (January 2020 to December 2020) and 2022 (January 2020 to June 2021) were linked publications of a single-center retrospective observational study. Among the 6 full-text RCTs, all were clearly randomized, with no evidence of selective reporting. None of the RCTs were blinded. Among the 11 full-text observational studies, 8 were rated as good quality and 3 as fair quality based on the NOS (Supplemental Table 2, available online at http://www.mcpiqojournal.org).

### Intervention Types

The interventions were classified into 4 different types based on their distinct features. Intervention types included patient visits (n=9),^17^^,^^20^^,^^21^^,^^24^^,^^27^^,^^30^^,^^31^^,^^34^, ^35^, ^36^ pharmacist integration (n=5),^16^^,^^18^^,^^19^^,^^22^^,^^23^^,^^33^ patient engagement or education (n=6),^29^^,^^32^^,^^37^, ^38^, ^39^, ^40^ and MDT or multispecialty team (MST) meetings without the patient being present (n=2).^25^^,^^26^^,^^28^

### Patient Characteristics

Although all studies included interventions targeting patients with at least 2 of 3 CKM conditions, some studies focused on 1 CKM condition while monitoring the other conditions. Only 3 studies included patients with 3 CKM conditions,^24^^,^^31^^,^^34^^,^^35^ 3 included patients with any 2 of 3 CKM conditions,^17^^,^^27^^,^^32^ and 16 focused on 1 CKM condition but also monitored the other 2 CKM conditions.

### Sample Size and Setting

The sample size of RCTs ranged from 25 to 2550. For observational studies, the sample size ranged from 14 to 9601. The mean age of patients ranged from 53 to 80 years, with the male population ranging from 35% to 100%. Only 7 studies reported mean body mass index, which ranged from 26.8 to 36.9 kg/m^2^, indicating that on average for these studies, participants were living with overweight or obesity. Four interventions were conducted in special populations (2 in US veterans,^20^^,^^22^ 1 in US underserved patients,^17^ and 1 in residents of rural British Columbia, Canada^32^). Kidney disease–related exclusion criteria, such as advanced CKD, estimated glomerular filtration rate of <45 mL/min/1.73 m^2^, or dialysis, were explicitly reported in 7 of the 22 interventions.^16^^,^^18^^,^^22^^,^^23^^,^^31^^,^^33^^,^^36^^,^^40^

### Interventions and Types

Interventions were grouped into 4 main categories by visit settings and types: outpatient specialty clinics (n=14), primary care practices (n=3), primary care plus multispecialty telemedicine consultation (n=3), and MDT meetings (n=2). Over 60% of interventions (14/22) were conducted in outpatient specialty clinics,^19^^,^^21^^,^^22^^,^^24^^,^^27^^,^^29^^,^^31^^,^^33^, ^34^, ^35^, ^36^, ^37^, ^38^, ^39^, ^40^ of which 3 involved virtual consultations,^19^^,^^21^^,^^27^ whereas the remaining 12 involved in-person visits including 3 interventions with additional telephone follow-up as well as in-person visits.^37^^,^^39^^,^^40^

Three of 22 interventions were conducted at primary care practices; 1 involved patient visits with telephone follow-up,^16^^,^^18^ the second involved patient access to a web-based platform and telephone consultations,^32^ and the third offered the option between in-person or video consultations.^23^ Three interventions involved primary care plus multispecialty telemedicine consultation with MDT where multispecialty distance sites provided consultations for patients at the primary care clinics.^17^^,^^20^^,^^30^ The remaining 2 interventions comprised MDT meetings; 1 involved virtual MDT/MST consultation meetings to optimize care for patients,^25^^,^^26^ whereas the other involved MDT/MST in-person meetings to reduce polypharmacy burden and adverse events in patients^28^ (Table 2).

Among the 22 interventions in the SLR, 12 specified a program lead who was responsible for coordinating the overall management of the intervention; 41% (9/22) of these leads were a pharmacist or a nurse. The other interventions were managed by a MDT, without specifying an intervention lead (Table 3). Eight of the 14 interventions conducted in outpatient specialty clinics reported a program lead (nurse-led=3, pharmacist-led=3, nephrologist-led=1, general practitioner-led=1), whereas the remaining 6 interventions in outpatient specialty clinics did not specify a program lead. All 3 interventions conducted in primary care practices specified a program lead (pharmacist-led=2, nurse-led=1). One of the 3 interventions involving primary care plus multispecialty telemedicine consultation with MDT was led by a nephrologist, whereas the other 2 interventions did not report a program lead. Similarly, the 2 interventions conducted through MDT meetings did not report a program lead.

**Table 3:** Outcomes, Grouped by Setting (n=22)

Reference, year (country)	Intervention/study name (NCT) and type	Intervention (No. of patients analyzed n) and follow-up	Comparator (No. of patients) and follow-up	Study population	Outcome summary: clinical	Outcome summary: HCRU and costs	Strengths/limitations
Outpatient specialist clinic (n=14 interventions in 15 publications)							
Al-Chalabi et al,^24^ 2022 (UK) (Congress abstract)	MRC clinic Type: Patient visits	n=209 Follow-up: NR	No comparator n: NA Follow-up: NA	All 3 CKM conditions	Use of guideline-directed medical therapies (RAAS inhibitors, SGLT2i, MRA, and β-blockers) was suboptimal in patients at referral to MRC clinic. Potassium binders were prescribed for 24 patients (11.5%) to facilitate optimization of RAAS inhibition and MRA use	HCRU: NR Costs: NR	Strengths: MRC service improved medication optimization and risk factor control Limitations: NR
Comaschi et al,^36^ 2020 (Italy) (protocol)	INSIDE study (protocol) Type: Patient visits	n=1500 (target) Follow-up: 2 y	Usual care n=1500 (target) Follow-up: 2 y	T2DM	Primary outcome: % of patients achieving target values of ≥2/3 risk factors (HbA1c, SBP, and LDL cholesterol) Secondary outcomes: MACE composite, number of early diagnoses of new-onset complications, adverse events, and safety	HCRU: NR Costs: Secondary outcomes: comparative cost analysis and cost-effectiveness analysis	Strengths: System based on a strong relationship between specialists and team Limitations: Use of different laboratories for examination measurements and no centralization of the samples
Chan et al,^40^ 2022 (Asia^a^)	A JADE RCT (NCT02176278) Type: Patient engagement/education	n: ITT population empowered: 802 Team-based empowered: 796 Follow-up: 12 mo	n: ITT population (usual care)=795 Follow-up: 12 mo	DKD	ITT analysis: The team-based empowered care group was more likely to attain multiple treatment targets than the usual care group (risk ratio, 1.17; 95% CI, 1.00-1.37) and the empowered care group (risk ratio, 1.25; 95% CI, 1.06-1.48) after adjustment for site	HCRU: NR Costs: NR	Strengths: Uses technology and nonphysician personnel to address unmet needs; results provide an implementation prototype to payers, policymakers, and HCP Limitations: Inclusion of patients with mild DKD limits generalizability to patients with advanced DKD; study population had good baseline risk factor control, suggesting healthy user bias; low adherence rates; few study sites had EHR
Dubrofsky et al,^31^ 2022 (Canada)	C.a.R.E. Clinic Type: Patient care	n=74 Follow-up: 27.7 (19.6) mo	No comparator n: NA Follow-up: NA	All 3 CKM conditions	Between last and first clinic visits: Significant improvement in LDL cholesterol (1.5 vs 1.9 mmol/L; *P*<.01) and HbA1c (7.1% vs 7.5%; *P*=.02) Increased proportion of patients with BP at target (52.7% vs 36.5%; *P*=.04) No change in BMI (29.6 vs 29.7 kg/m^2^; *P*=.15) Higher uptake of guideline-directed medical therapies including statins, last vs first visit: (93.2% vs 81.1%; *P*=.01), SGLT2i (35.1% vs 4.1%; *P*<.01), and GLP-1RA (13.5% vs 4.1%; *P*=.02); RAAS inhibitor use was already high at baseline (81.8% vs 78.4%; *P*=.56)	HCRU: NR Costs: NR	Strengths: MDT clinic provides cohesive patient care to optimize evidence-based targets, facilitates clinical trial recruitment, and provides unique training opportunities Limitations: NR
Jegatheesan et al,^29^ 2022 (Australia) (Congress abstract)	LANDMARK III (multidisciplinary lifestyle intervention) Type: Patient engagement/education Patient visits	n: NR Follow-up: 3 y	Usual care n: NR Follow-up: 3 y	CKD	Patients treated with usual care had a significantly greater decline in global longitudinal strain than patients in the intervention group, who had preservation of global longitudinal strain (−1.1% vs −0.4%; *P*<.001). There were no significant changes in EF, LV mass index, LV filling pressure, fractional shortening, or relative wall thickness between groups	HCRU: NR Costs: NR	NR (congress abstract)
Keel et al,^34^ 2020 (Sweden)	CareHND (NCT03362983) Type: Patient visits	n=314 Follow-up: NR	No comparator n: NA Follow-up: NA	All 3 CKM conditions	NA	HCRU comprised 7 outpatient care delivery activities. The annual count was the highest for nurse telephone consultations (1545), followed by team visits (478), nurse visits (278), team conference (240), physician telephone consultation (159), physician visit (151), and new visits (143). Costs: Total annual cost of the HND center was €471,791. Team visits accounted for most of the cost (€369/visit = €176,567), followed by nurse telephone consultations (€211/call = €78,286), and new visits to the HND center (€367/visit = €52,528). Other annual costs were physician visits (€44,780), team conferences (€27,470), uncaptured capacity (€24,138), physician telephone consultations (€9469), and nurse visits (€5854)	Strengths: Based on the cost analysis, hospital administrators selected the HND center for ongoing development Limitations: Cost estimates may vary from year to year (see Rafiq et al^35^ for additional details)
Rafiq et al,^35^ 2019 (Sweden)	CareHND (NCT03362983) Type: Patient visits	n=42 Follow-up: 6 mo	Usual care n=35 Follow-up: 6 mo	All 3 CKM conditions	NR	HCRU: Mean telephone visit frequencies increased the intervention arm from baseline (1.2 vs 1.0; *P*=.001) but decreased in the control arm (0.9 vs 1.0; *P*=.001). The remaining care utilization variables (eg, length of stay, inpatient visits, and emergency department visits) did not differ from baseline in either arm. Costs: NR	Strengths: Initiatives like the HND center may positively influence health care utilization; patients obtain all necessary treatments at a single location Limitations: HND patients consume large amounts of health care resources. Delivering integrated care to treat complex conditions (59 separate diagnoses) is a huge task for providers
Li and Radhakrishnan,^19^ 2021 (USA)	Pharmacist–physician collaborative care Type: pharmacist integration	n=92 Follow-up: median (range): 3 mo (0.5-17 mo)	No comparator n: NA Follow-up: NA	CKD	Frequent follow-ups with pharmacists helped patients to remain engaged in disease management Significant increase in number of patients meeting treatment targets In patients with ≥2 pharmacy visits, achievement of diabetes targets increased from 23% to 67% and HTN from 29% to 58% Patients with pharmacist interventions plus a technology tool (eg, utilization of patient portal) achieved a higher goal attainment rate for HTN than those with pharmacist intervention but no technology tool (73% vs 50%)	HCRU: NR Costs: NR	Strengths: NR; however, pharmacist–physician collaboration presents an opportunity to optimize care for patients with CKD; technology tools (remote BP monitoring) enhanced patient engagement and improved goal achievement Limitations: NR
Lim et al,^39^ 2020 (Hong Kong)	Technology-assisted integrated care Type: Patient engagement/education	n: JADE: 9601 JADE-P: 3436 Follow-up: median (IQR): 6 y (4.2-7.0 y)	Non-JADE n=3587 Follow-up: median (IQR): 6 y (4.2-7.0 y)	T2DM	The non-JADE group (publicly funded evaluation only) had 19%-34% higher risk of clinical events, including hospitalization, than the JADE group (publicly funded evaluation with JADE reports + group education). Compared with the JADE group, the JADE-P group (self-paid evaluation with JADE reports + personalized empowerment + annual telephone reminder) had 23%-36% lower risk of clinical events, including hospitalization and death	HCRU: NR Costs: NR	Strength: JADE provided efficient use of ICT and nonphysician personnel and used data-driven personalized self-management tools to improve outcomes in both public and private health care settings Limitation: No access to medical records from private sector (but patients expected to still have some public sector records). JADE-P group may be using private service, so study could have missed major events treated in private sector
Narain et al,^27^ 2022 (UK) (congress abstract)	CMC Type: Patient visits	n=174 Follow-up: NR	No comparator n: NA Follow-up: NA	Any 2 of 3	Antidiabetic drugs were initiated/titrated in 107 patients, mean HbA1c reduced in 40/107 patients (−18 mmol/mol), but mean HbA1c increased in 14 patients (+7 mmol/mol). Weight loss was reported in 18/88 patients who initiated or optimized SGLT2i, 7/27 on metformin, and 12/19 on GLP-1RA. SGLT2i were stopped in 3 patients due to intolerance/contraindication. Of 103 patients on SGLT2i, 8 had further HF-related hospitalizations	HCRU: Patients were referred to other clinicians/services, including dietetics (n=13), diabetes specialist nurse (n=14), endocrine consultants (n=9), cardiorenal clinics (n=11), renal diabetes clinics (n=5), renal (n=2), and HF nurse specialists (n=10) Costs: NR	Strengths: NR; however, intervention was achieved virtually due to the COVID-19 pandemic (provides evidence to support virtual approaches) Limitations: Short follow-up time
Neeland et al,^21^ 2022 (USA)	CINEMA program Type: Patient visits	n=113 Follow-up: 1 y	No comparator n: NA Follow-up: NA	T2DM	Patients had significant reductions from baseline in glycosylated hemoglobin (−10.8%), total cholesterol (−7.9%), LDL cholesterol (−13.5%), SBP (−4.0%), and BMI (−2.7%); *P*≤.001 for all. SGLT2i and GLP-1RA prescription rates increased from baseline to first follow-up visit, from 37% to 88% (all enrolled patients)	HCRU: NR Costs: NR	Strengths: NR, but program enabled patients to see whole care team in single visit, reducing time burden Limitations: Intervention was in a single academic health system, so may not be generalizable to other settings; follow-up was short (∼3 mo) and outcomes such as MACE are rare; no assessment of providers’ views of program goals and outcomes; unable to evaluate full implementation because data focused on initial experience of the program
Schutze et al,^33^ 2021 (Germany)	NR (adding clinical pharmacist to the therapeutic team) Type: pharmacist integration, HCP training/education	n=96 Follow-up: 6 mo	Preintervention care n=160 Follow-up: 6 mo	CKD	The number of drug-related problems decreased significantly in the intervention group compared with that in control (*P*<.001 for all subgroups between recruitment and 6 mo)	HCRU: NR Costs: NR	Strengths: The interventional tools increased GPs’ acceptance of prescribing recommendations and compliance with specialist recommendations; comprehensive medication reviews by pharmacists led to higher detection rates of poor adherence, unsuitable therapies, and adverse events Limitations: NR
Tan et al,^37^ 2019 (Singapore) (protocol)	IDEALS (NCT03413215) (protocol) Type: Patient engagement/education	n=25 Follow-up: 3 y	Usual care n=25 Follow-up: 3 y	DKD	Potential clinical outcomes Primary outcomes: CV events, rate of progression of nephropathy, and development of ESKD Secondary end points: Proportions of patients with documented improved control of CV risk factors (HbA1c, BP, LDL cholesterol, and weight loss), frequency of hypoglycemia, changes in femoral intima-media thickness Other outcomes: Prevalence of peripheral atherosclerosis and utility of lower extremity arterial ultrasound to predict cardiocerebrovascular events, amputation, and peripheral intervention	HCRU—secondary outcome: number of hospitalization days Costs: NR	Strengths: NR; however, provide a model for developing a patient-centric program that integrates HCPs to optimize resource allocation; incorporate health literacy and counseling, as well as personalized educational materials Limitations: NR
Triantafylidis et al,^22^ 2021 (USA)	NR (embedding clinical pharmacist within interprofessional nephrology clinic) Type: Pharmacist integration	n=14 Follow-up: 6 mo	No comparator n: NA Follow-up: NA	CKD	Mean % UACR improved (−12%±61%), with mean change greater in patients with higher baseline UACR. Mean change in HbA1c was modest (0.3%±0.6%). Empagliflozin initiation required changes to diabetes, HTN, and diuretic regimens in 13 patients (93%), but no serious adverse drug reactions occurred	HCRU: NR Costs: Over 3 mo, each patient required 4.8 h of pharmacist time × 14 patients = 67 h, at $63/h (2017 national average hourly rate) = $4221 for 14 patients over 3 mo. The program could save an estimated $9300/y (2010 US dollars) by preventing one’s progression of CKD stage 3 to stage 4, with additional cost savings by preventing hypoglycemia requiring ER visits, HF admissions, and ESKD	Strengths: The interprofessional model provided safe and effective empagliflozin initiation in real-world patients with DKD and complex medical conditions Limitation: Study period (6 mo) may not have been long enough to detect meaningful improvements in HbA1c, as seen in other programs
Wongprasert et al,^38^ 2022 (Thailand) (congress abstract)	MDT Care + patient empowerment (NCT: NR) Type: Patient engagement/education	n=25 Follow-up: 2 y	Usual care n=25 Follow-up: 2 y	DKD	Composite of T2DM-related complications and all-cause mortality: 21.2% in intervention group vs 40.6% in usual care. Patients receiving intervention vs usual care had significantly greater reductions in CVD risk by 52.6% (95% CI, 50.5-54.6), microvascular complications by 10.9% (95% CI, 6.8-14.6), mortality by 65.1% (95% CI, 61.3-62.9), specialist visits by 31.0% (95% CI, 30.6-32.4), emergency attendance by 39.2% (95% CI, 36.8-40.5), and hospitalizations by 53.5% (95% CI, 53.2-56.7). Patients with low baseline CVD risk benefited most from the intervention: CVD and mortality risk decreased by 58.4% (95% CI, 50.8-64.5) and 79.6% (95% CI, 72.3-81.0), respectively	HCRU: NR Costs: NR	NR (congress abstract)
Primary care practices (n=3 interventions in 4 publications)							
Anderegg et al,^16^ 2018 (USA)	CAPTION trial (NCT00935077) Type: pharmacist integration	n=335 Follow-up: 9 mo	Usual care n=108 Follow-up: 9 mo	HTN	Pharmacist integration resulted in better BP control than usual care; mean SPB reduction in mm Hg (95% CI): 8.64 (−12.8, 4.49; *P*<.001) DBP reduction: 2.90 (−5.55, −0.25; *P*=.0323) BP goals Seventh Report of the Joint National Committee on Prevention, Detection, Evaluation, and Treatment of High Blood Pressure analysis (adjusted OR, 1.97 [95% CI, 1.01-3.86]; *P*=.0470) 2014 guideline analysis (adjusted OR, 2.16 [95% CI, 1.21-3.85]; *P*=.0102)	HCRU: NR Costs: NR	Strengths: Pharmacists had flexibility in how they optimized BP control; intervention was effective across minority groups, income levels, and insurance type Limitations: 15% of patients did not complete the follow-up visit
Dixon et al,^18^ 2021 (USA)	CAPTION trial (NCT00935077) Type: pharmacist integration	n: patients with T2DM or CKD 9-mo intervention: 86 24-mo intervention: 100 All patients 9-mo intervention: 169 24-mo intervention: 180 Follow-up: 9 mo; 24 mo	Usual care n: patients with T2DM or CKD: 9-mo intervention: 84 24-mo intervention: 40 All patients: 175 Follow-up: 9 mo; 24 mo	HTN	Patients with T2DM or CKD At 9 mo, % TTR for SBP, median (IQR) 9-mo intervention: 27.9 (3.9-50.7), N=86 24-mo intervention: 6.0 (0-43.8), N=100 Combined intervention: 20.2 (0-47.7), N=186 Usual care: 0 (0-25.1), N=84 *P*=.0003 (9 vs 24 mo vs usual care); *P*=.0011 (combined vs usual care) At 24 mo, % TTR for SBP, median (IQR) 9-mo intervention: 37.8 (25.3-63.1), n=62 24-mo intervention: 29.0 (12.4-49.8), n=92 Combined intervention: 34.0 (16.0-54.5), n=154 Usual care: 20.3 (0-56.8), n=40 *P*=.0060 (9 vs 24 mo vs usual care); *P*=.0217 (combined vs usual care)	HCRU: NR Costs: NR	Physician/pharmacist collaborative care model achieved better control of SBP (longer TTR for SBP and shorter time to first TTR) than usual care in patients with HTN, particularly those with CKD or T2DM, leading to lower risk of CV events
Lear et al,^32^ 2021 (Canada)	iCDM (NCT01342263) Type: Patient engagement/education Behavioral change	n=116 Follow-up: 24 mo	Usual care n=113 Follow-up: 24 mo	Any 2 of 3	Significantly fewer patients in iCDM group vs usual care had ≥1 ACH (32/116 [27.6%] vs 46/113 [40.7%]; OR, 0.55; 95% CI, 0.31-0.96; *P*=.03. iCDM group had lower odds of composite outcome of ACH or death (37/116 [31.9%] vs 51/113 [45.1%]; OR, 0.57; 95% CI, 0.33-0.98; *P*=.04) and had a lower risk of time to first hospitalization than usual care group (hazard ratio, 0.62; 95% CI, 0.39-0.97; *P*=.04) 25 fewer ACH occurred in the iCDM group vs usual care (56 hospitalizations vs 81 hospitalizations; 30.9% reduction) but was not statistically different (relative risk, 0.68; 95% CI, 0.43-1.10; *P*=.12) after adjusting for age, sex, and number of chronic conditions 229 fewer in-hospital days occurred in the iCDM group vs usual care (282 vs 511 d) but did not differ significantly (relative risk, 0.52; 95% CI, 0.24-1.10; *P*=.09)	HCRU: NR Costs: NR	Strengths: High uptake of the intervention, which was integrated with and informed by primary care. Remote communication enabled patient monitoring across geographical regions Limitations: NR
Vu et al,^23^ 2022 (USA)	Positive Kidney Health Type: Pharmacist integration	n=20 Follow-up: NR	No comparator n: NA Follow-up: NA	CKD	Of 9 patients with new microalbuminuria, 2 were started on renoprotective medications; patients had improved understanding of their kidney function test results (serum creatinine, eGFR, and UACR) T2DM medication was intensified for 3/10 patients with T2DM; SGLT2i was recommended for 4 patients (but only initiated in 2 patients) 2 patients smoked cigarettes; both participated in smoking cessation 3 patients received counseling for chronic NSAID use and switched to acetaminophen or topical diclofenac	HCRU: NR Costs: NR	Strengths: Telehealth options were convenient for patients who could connect via online video; pharmacists were able to engage patients in learning the importance of monitoring and self-management of kidney health; pharmacists helped increase kidney function screening Limitations: Barriers to enrollment/low uptake from PCPs; restriction on pharmacist interventions without a collaborative practice agreement; PCPs not always aware of benefits/need of SGLT2i for DKD; program targeted less medically optimized patients requiring complex case management; return visits 2-3 were a challenge for some participants
Primary/telemedicine with MDT (n=3 interventions in 3 publications)							
Burke et al,^17^ 2023 (USA)	NR (telemedicine between primary care site and multispecialty distance site) Type: Patient visits	n=104 Follow-up: NR	In-person specialist care n=199 Follow-up: NR	Any 2 of 3	HbA1c decline from preintervention to intervention period was larger in telemedicine group (mean decline, telemedicine=1.5 vs controls=0.4; *P*_trend_=0.053). No significant difference in mean arterial pressure (*P*=.26), serum creatinine (*P*=.90), and eGFR (*P*=.56) was found between the 2 groups in the change from preintervention to intervention periods	HCRU: No difference in no-show rate between the 2 groups (adjusted OR, 1.03 [0.66-1.63]; *P*=.87). Patients’ satisfaction was higher in telemedicine group than that with controls (overall satisfaction in telemedicine group rated between agree and strongly agree) Costs: NR	Strengths: The intervention reduced transportation barriers and improved patient engagement Limitations: Lack of sufficient standard in-person visit data for specialists, limited knowledge of telemedicine (study conducted before pandemic), which may have decreased engagement in telespecialty consultation; implementation of telemedicine program may exacerbate existing disparities in access and/or outcomes
Katz et al,^30^ 2018 (Australia)	iConnect CKD Type: Patient visits	n=61 Follow-up: 12 mo	In-person clinic n=9 Follow-up: 12 mo	CKD	At 12 mo, there was no difference in outcomes between patients with virtual consultation and face-to-face consultation. Of the 23 high-risk patients (14 followed up virtually and 9 face-to-face), there was no statistically significant difference in eGFR or albuminuria at 12 mo for virtual consultation vs face-to-face clinics, but high-risk face-to-face patients had significantly higher UACR at 6 mo than virtual consultation patients. 5 patients received erythropoietin (via the virtual consultation) for renal anemia	HCRU: NR Costs: NR	Strengths: Web-based software to track patients’ progress, GPs interested in decision support and advice; virtual consult model successfully interfaced with the GP, primary care, and patient; virtual consultation maintains important primary relationship between GP and patient Limitations: Obstacles in system structure (old and cumbersome software/time consuming), some GPs reluctant to be involved in long-term follow-up (just wanted decision support); GPs refer patients who do not fit eligibility criteria
Lu et al,^20^ 2021 (USA)	V-IMPACT Type: Patient visits	n=4505 Follow-up: 1 y	In-person care n=4505 Follow-up: 1 y	T2DM	Between the V-IMPACT and control group, there was no significant difference in change in HbA1c or in % of patients with controlled BP (both the <140/90- or 130/90-mm Hg threshold). There were significantly larger increases in patients prescribed a statin or a ACEi/ARB, as well as testing for macroalbuminuria in the V-IMAPCT group	HCRU: NR Costs: NR	Strengths: Telemedicine increased communication between primary care and specialists; allows underserved patients to receive optimized care without travel; and reduces the supply gap in rural areas Limitations: Implemented within a Veterans Affairs health system (higher proportion of men and patients with multimorbidity than the general US population), so limits generalization
MDT meetings (n=2 interventions in 3 publications)							
Essa et al,^25^ 2021 (UK) (congress abstract)	NR (HF multispecialty MDT virtual meetings) Type: MDT meetings (virtual)	n=189 Follow-up: Median (range): 6 mo (1-13 mo)	Preintervention care n=189 Follow-up: Median (range): 6 mo (1-13 mo)	HF	NR	HCRU: the mean number of hospitalizations was significantly reduced post-MDT (0.7 vs 0.2; *P*<.01); saving 730 bed-days. The mean (SD) number of outpatient clinic visits avoided was 1.7 (0.4), and the MDT prevented 277 clinic appointments (convenient for patients and cost saving) Costs: total costs for the MDT meetings were £15,400 and the clinic appointments they generated (31) cost ∼£3720. MDT meetings saved £33,352 in avoided clinic visits (n=277) and ∼£260,000 from reduced hospitalizations (avoided bed-days=730)	Strengths: NR, but a virtual MDT approach would provide seamless integration between primary and secondary care Limitations: NR (see Essa et al,^26^ 2022)
Essa et al,^26^ 2022 (UK)	NR (HF multispecialty MDT virtual meetings) Type: MDT meetings (virtual)	n=334 Follow-up: 13.9 (4) mo	Preintervention care n=334 Follow-up: 13.9 (4) mo	HF	Pre-MDT vs post-MDT, patients with comorbid CKD had an increase in ACEi/ARB initiation (9% vs 71%; *P*<.001) and quadruple therapy (46% vs 71%; *P*<.0001). More patients were prescribed potassium binders post-MDT (2 vs 13) and SGLT2i addition was recommended in 91 patients. Mean (SD) HbA1c levels significantly improved pre-MDT vs post-MDT (68 [11] vs 61 [9] mmol/mol; *P*<.001). Deprescribing to reduce anticholinergic burden was significant (*P*<.001), likely leading to reduced hospital admissions (*P*=.003). Owing to MDT consensus recommendations, 16% patients underwent ICD or CRT/CRTD therapy and 3% were referred for transplant assessment, but there was no difference pre-MDT vs post-MDT; 5% of patients were referred to dialysis assessment clinics	HCRU: ACH decreased significantly from 371 (4126 bed-days) pre-MDT to 205 post-MDT (2540 bed-days). Hospitalizations per person: pre-MDT vs post-MDT, 1.1 ± 0.4 vs 0.6 ± 0.2; *P*<.001). There was a 36% reduction in HF hospitalizations (*P*=.03) and 55% in non-HF hospitalizations (*P*=.04). The number of outpatient clinic attendances decreased post-MDT, from 946 (pre-MDT) to 465 (post-MDT). There was a 51% reduction in HF appointments (481 vs 242), and 55% reduction in non-HF appointments (465 vs 223). Costs: Cost per MDT meeting was £1800, totaling £32,400 over the study duration (Jan 2020-Jun 2021). MDT meeting recommendations saved 534 clinic appointments, resulting in a cost saving of £80,100. The reduction in hospitalizations saved £634,400 from 1586 avoided bed-days. Overall, total cost saving of MDT was £664,550	Strengths: Virtual MDT limits the need for face-to-face visits; enables holistic care targeted to HF and comorbidities together; virtual MDT saves time, costs, and interspecialty referrals/clinic visits; benefits frail patients with HF Limitation: virtual MDT approach has the potential for miscommunication (lack of patient presence); requires robust service planning to ensure attendance by all team members in 1 meeting
Saied et al,^28^ 2022 (UK) (congress abstract)	NR (HF multispecialty MDT) Type: MDT/MST meetings	n=148 Follow-up: NR	No comparator n: NA Follow-up: NA	HF	The multispecialty HF MDT reduced polypharmacy burden in patients with 6-10 medications (median, 1 medication; range, 0-2) and in patients with >10 medications (median, 3 medications; range, 0-5)	HCRU: NR Costs: NR	Strengths: NR; however, the MST/MDT helped reduce the number of medications in patients with HF Limitations: NR

Publications highlighted in similar colors represent the same interventions.

ACEi, angiotensin-converting enzyme inhibitor; ACH, all-cause hospitalization; ARB, angiotensin receptor blocker; BMI, body mass index; BP, blood pressure; CAPTION, Collaboration Among Pharmacists and Physicians to Improve Outcomes Now; C.a.R.E. Clinic, Cardiac and Renal Endocrine Clinic, Toronto General Hospital; CI, confidence interval; CINEMA (program), Center for Integrated and Novel Approaches in Vascular-Metabolic Disease; CKD, chronic kidney disease; CMC, cardiometabolic clinic; CRT, cardiac resynchronization therapy; CRTD, cardiac resynchronization therapy device; CV, cardiovascular; CVD, cardiovascular disease; DKD, diabetic kidney disease; EF, ejection fraction; eGFR, estimated glomerular filtration rate; EHR, electronic health record; ER, emergency room; ESKD, end stage kidney disease; GLP-1RA, glucagon-like peptide-1 receptor agonist; GP, general practitioner; HbA1c, glycated hemoglobin; HCP, health care professional; HCRU, health care resource utilization; HF, heart failure; HND, Heart Nephrology Diabetes (center); HTN, hypertension; ICD, implantable cardioverter-defibrillator; iCDM, Internet-based Platform for Managing Chronic Diseases; ICT, information and communications technology; IDEALS, Integrated Diabetes Education, Awareness and Lifestyle modification in Singapore; INSIDE (study), Integration of care for reaching targetS In Diabetic patiEnts; IT, information technology; ITT, intent-to-treat; IQR, interquartile range; JADE, Joint Asia Diabetes Evaluation; LDL, low-density lipoprotein; LV, left ventricle; MACE, major adverse cardiovascular event; MDT, multidisciplinary team; MRA, mineralocorticoid receptor antagonist; MRC, Metabolic-Renal-Cardiac (clinic); MST, multispecialty team; NA, not applicable; NCT, national clinical trial; NR, not reported; NSAID, nonsteroidal anti-inflammatory drugs; OR, odds ratio; PCP, primary care physician; RAAS, renin-angiotensin-aldosterone system; RCT, randomized controlled trial; SBP, systolic blood pressure; SGLT2i, sodium-glucose cotransporter 2 inhibitor; T2DM, type 2 diabetes mellitus; TTR, time in target range; UACR, urine albumin-creatinine ratio; UK, United Kingdom; USA, United States of America; V-IMPACT, Virtual Integrated Multisite Patient Aligned Care Team.

a 8 countries or regions in Asia: China, Hong Kong, Malaysia, Philippines, South Korea, Taiwan, Thailand, Vietnam.

Program effectiveness was reported through clinical effectiveness (n=19), cost effectiveness (n=4), and health care resource utilization (n=2) (Supplemental Table 3, available online at http://www.mcpiqojournal.org). Several interventions were designed to provide coordinated care for populations who were likely to have difficulty accessing multispecialty care for CKM conditions, including urban underserved populations,^17^ rural populations,^32^ and US Veterans.^20^^,^^22^ Two protocol-only publications were also included in this SLR because they provided details of proposed coordinated care programs.^36^^,^^37^ Seven of the 25 studies mentioned use of glucagon-like peptide-1 receptor agonists (GLP-1RA) and sodium-glucose cotransporter 2 inhibitors (SGLT2i) (Table 3).^21^, ^22^, ^23^, ^24^^,^^26^^,^^27^^,^^31^

### Benefits of Coordinated Care Programs

Effective coordinated care programs may positively impact patient satisfaction and continuity of care, resulting in improved patient outcomes while decreasing health care costs versus usual care (Supplemental Figure 1, available online at http://www.mcpiqojournal.org). All coordinated care programs included in this SLR except CareHND^34^^,^^35^ and the 2 protocols^36^^,^^37^ reported clinical effectiveness, and therefore improved patient wellbeing, with a high proportion of patients receiving coordinated care achieving therapeutic target values (eg, cholesterol, systolic blood pressure, and glycated hemoglobin). Among the RCTs, patients in team-based intervention groups reported a smaller decline in a heart function measure (global longitudinal strain by echocardiography)^29^; reported greater reductions in cardiovascular disease events, hospitalizations, and mortality rates^32^^,^^38^; and were more likely to attain multiple treatment targets^40^ than those treated with usual care. Similarly, in the interventions reporting resource utilization, the CKM coordinated care programs effectively reduced health care resource utilization and costs because of fewer hospitalizations and shorter hospital stays. Two different types of coordinated care programs reported lower costs: programs incorporating virtual or telephone consultations and patient engagement or education sessions delivered by nurses or pharmacists. An additional benefit of the nurse or pharmacist-delivered interventions was empowering patients to better manage their conditions.

Pharmacist–physician collaboration was effective in optimizing care for patients with CKM conditions. Through regular medication reviews, pharmacists were able to identify unsuitable therapies and treatment-related adverse events as well as address adherence issues. Multispecialty telemedicine consultations not only improved communication between primary care practitioners and clinical specialists^25^^,^^26^ but also increased access to specialist care for underserved patients.^17^ Moreover, multispecialty telemedicine consultations reduced travel barriers and enhanced patients’ involvement in their care.^17^

### Challenges of Coordinated Care Programs

Studies reported program-related challenges (Supplemental Table 3). The main reported challenge of patient engagement or education interventions was nonattendance for return visits, particularly when multiple training sessions were required.^23^ Moreover, some patients had difficulty attending health care clinics for physical examinations or in-person sessions.^17^ Some patients and primary care practitioners experienced problems with information technology integration for virtual programs owing to either system complexity or a lack of access to equipment.^30^ In another study, the authors were limited to patient self-reports for monitoring, medication profile, and side effects without the ability to confirm the accuracy or completeness of these records.^33^ In addition, gaining consensus among interested parties for program protocols and care management was a challenge, which hindered effective program implementation.^21^

## Discussion

Despite the call for coordinated CKM care by several guidelines and substantial advancements in treatment of individual CKM conditions, the lack of the implementation and integration of holistic approaches to CKM care in clinical practice is still apparent.^12^ This SLR provides an assessment of current coordinated care programs and describes the types, components, and effectiveness of programs used to treat, evaluate, and educate patients with CKM syndrome, as well as program strengths, limitations, and implementation challenges. Twenty-two interventions, conducted in 15 countries across 4 continents, met study inclusion criteria. We categorized the interventions into 4 main types: patient visits, patient engagement or education, pharmacist integration, and MDT/MST meetings. The overall objective of all interventions was to improve health-related outcomes for patients, although mode of delivery varied. Overall, results from the included coordinated care programs showed improvements in several outcomes including higher patient satisfaction and fewer health-related problems.

The AHA has called for the use of MDTs to reduce the fragmentation of care for patients with CKM syndrome and recommends that MDTs include representation from primary care, cardiology, nephrology, endocrinology, pharmacy, and nursing, as well as care navigators, social workers, or community health workers.^3^ Indeed, several of the programs identified in this SLR contained both MST and MDT to provide coordinated care. Among the 22 interventions in the SLR, 12 specified a program lead, of which 45% were led by a pharmacist or by a nurse. Designated leadership and an understanding of team roles and responsibilities are important for successful program implementation. Programs in heart failure and T2D instituting a specific team leader demonstrated that this approach could help optimize guideline-directed medical therapy by overcoming time and resource constraints typically faced by other clinicians.^41^^,^^42^ Similar to our results, an SLR of 33 studies found an increase in guideline adherence in pharmacist-led interventions for patients with atrial fibrillation among programs that utilized coordinated care models.^43^

Of the 25 included publications, 7 mentioned use of GLP-1RA and SGLT2i. These therapies have demonstrated to be beneficial in the management and prevention of cardiovascular disease events and CKD progression and are featured in CKM treatment guidelines.^3^ Variation in the use of GLP-1RA and SGLT2is in the included studies may have resulted in heterogeneity in cost and clinical outcomes assessment across studies.

Advances in digital health and telehealth offer advances in management of various chronic conditions. Our results align with findings that integrated telehealth improved management of chronic diseases and reduced hospitalization and mortality.^44^ However, patient and clinician access to technology resources is essential for the success of telehealth programs. Additionally, a meta-analysis of 72 studies of the effects on cardiovascular outcomes of introducing telehealth found a significant reduction in cardiovascular disease–related mortality and hospitalization for cardiovascular causes.^45^ Another SLR of interventions in patients with cardiovascular disease and hypertension concluded that telehealth was comparable with in-person care but offered benefits by increasing opportunities for engagement, communication, and monitoring outside a clinical setting.^46^ Telehealth has been shown to be effective in short-term management of patients with diabetes.^47^ It also has the potential to increase health care use in groups who are often excluded from standard models of care.^48^ In our SLR, programs that included virtual consultations or telehealth also showed benefits for attendance rates and health care costs.

The 22 interventions in this SLR varied in their objectives and design. Although the interventions treated or monitored CKM conditions, the target population varied by the intervention, with only 3 specifically targeting patients with 3 CKM conditions.^24^^,^^31^^,^^34^^,^^35^

### Strengths and Limitations

The main strength of our review lies in its use of a standardized, comprehensive, and transparent approach to identify and quality-appraise studies. This SLR was conducted according to PRISMA guidelines and included a thorough and comprehensive literature search to identify relevant studies. In addition, the study selection was completed by 2 independent investigators. Moreover, to our knowledge, this is the first SLR reporting the types and effectiveness of coordinated care programs for patients with CKM conditions; additionally, no prior SLR on coordinated care included all 3 CKM conditions. Our SLR provides evidence that coordinated care addressing CKM conditions is associated with overall better clinical outcomes, lower health care resource utilization, and reduced health care costs.

This SLR also has limitations. First, there was considerable heterogeneity in intervention design, delivery, population, and outcomes assessed. For example, some interventions required participants to attend the clinic, whereas some used telehealth and others comprised MDT meetings only. Similarly, some interventions treated 3 CKM conditions, whereas others treated only 1 condition but referred the patients to other specialists for other CKM conditions. Furthermore, 24 articles were excluded from the SLR because the intervention did not address the CKM conditions. In addition to heterogeneity, few studies reported program-related challenges or what components were responsible for positive results. As such, specific recommendations for successful implementation of coordinated care programs are not possible. On the contrary, it is also unlikely that a single type of program would be applicable to all settings. Second, the follow-up period varied for the interventions, with some reporting short follow-up periods as an important limitation in assessing program outcomes. Moreover, patients not returning for follow-up visits also limited outcome assessment. Owing to the heterogeneity of the coordinated care programs and the differences in evaluation criteria, specific estimates of the evidence were not assessed, rather the overall effectiveness of the programs were summarized. Finally, selection bias cannot be eliminated because only English-language studies were eligible for inclusion. Furthermore, publication bias is very likely because some coordinated care programs were likely not published because of their lack of effectiveness. Although no restriction was placed on sample size, we limited the period to studies published from January 2015 (to correspond with early cardiovascular outcome trial availability) to March 2023 and congress abstracts published from January 2021 to March 2023, so studies published after that date would not have been included. In addition, because this SLR only searched Medline, Embase, and Embase congress abstracts, relevant studies not indexed in these databases could have been missed.

## Conclusion

This SLR shows that coordinated care for patients with CKM conditions improves health-related outcomes and reduces health care costs. Integrating pharmacists into the care team has proven effective in identifying medication adverse events, educating patients, and optimizing medication management. In addition, telehealth options and MDT consultations improved access to CKM care, which may be particularly useful in limited-access or remote areas. Interventions that included patient education and engagement also increased self-management. Future research is needed to develop coordinated care programs with standard reporting, assessment of effectiveness with contemporaneous controls and to identify components that are most beneficial along with implementation methods. Broader acceptance and incorporation of coordinated care models in clinical practice is key for improved care for patients and to reduce the burden of primary and secondary care. Barriers for the implementation of coordinated CKM care include role delineation, care integration, and resource constraints for care delivery.^49^

## Potential Competing Interests

Dr Duru reports research grants/contracts with 10.13039/100000030Centers for Disease Control and Prevention, 10.13039/100000002National Institutes of Health, 10.13039/100000052NIH Office of the Director, Patient-Centered Outcomes Research Institute, and Terasaki Institute of Biomedical Innovation. Dr Alicic reports NIH research grants (OT2HL161847, OT2OD032581, and U24TR001608) and CDC project (number 75D301-21-P-12254); other support from Travere Therapeutics Inc, outside of the submitted work, and personal fees from Boehringer Ingelheim; other support from 10.13039/100004326Bayer AG, 10.13039/100004325AstraZeneca, 10.13039/501100004191Novo Nordisk, 10.13039/501100021096The George Institute for Global Health, and CareDx Inc. Dr Vaduganathan reports research grant support, served on advisory boards, or had speaker engagements with American Regent, Amgen, AstraZeneca, Bayer AG, Baxter Healthcare, BMS, Boehringer Ingelheim, Chiesi, Cytokinetics, Lexicon Pharmaceuticals, Merck, Novartis, Novo Nordisk, Pharmacosmos, Relypsa, Roche Diagnostics, Sanofi, and Tricog Health; and participation in clinical trial committees for studies sponsored by AstraZeneca, Bayer AG, Galmed, Impulse Dynamics, Novartis, and Occlutech. Dr St. Peter reports NIH research grant (1R01DK124333-01A1), a grant from the American College of Clinical Pharmacy, United States Renal Data Systems Coordinating Center contract (HSN 75N94019C00006), contract from Chronic Disease Research Group; is the director for Advancing Kidney Health through Optimal Medication Management initiative; and reports consultancy fees from Bayer, Boehringer Ingelheim/Lilly, Fresensius Medical Care, and GSK. Roberts reports NIH research grants (5U24DK114886-07 and 5U01DK133090-02); consultancy fees from Maze Therapeutics, University of Minnesota Office of Discovery and Translation “Reduce Medication-Related Disparities in African American Patients with Chronic Kidney Disease” research project, Advancing Kidney Health through Optimal Medication Management (AKHOMM), and Critical Path Institute; honoraria for serving on the patient advisory boards of ProKidney, the APOLLO APOL1 Long-term Kidney Transplantation Community Advisory Council (CAC), Kidney Precision Medicine Project Community Engagement Committee, the TwoPlus patient advisory board, the Critical Path Institute Drug-Induced-Kidney-Injury patient engagement committee and BioMarker Data Repository (BMDR) Governance Committee, Mount Sinai – Center for Kidney Disease Innovation Patient Advisory Committee, External Expert Panel for the Chronic Renal Insufficiency Study (CRIC), New York University Xenotransplantation Working Group, the University of Washington Kidney Research Institute Patient Advisory Committee, American Heart Association CKM Patient Advisory Group, Kidney Health Initiative Board of Directors, and ASN for the Celeste Castillo Lee presentation. Dr Rangaswami reports consultancy fees from AstraZeneca, Boehringer Ingelheim/Eli Lilly and Company, and Edwards Lifesciences and participation in advisory board of Procyrion Inc (Aortix). Dr Nicholas reports research support from Terasaki Institute of NIH/NCATS, NIH/NIMHD, Bayer, Biomedical Innovation, CDC, Goldfinch Bio, and Travere; consulting fees from AstraZeneca, Bayer, Boehringer Ingelheim/Lily Pharmaceuticals, Gilead Sciences Inc, Janssen Pharmaceutical, Novo Nordisk, and Vifor; national leadership roles in Bayer, Gilead Sciences Inc, and Janssen Pharma; advisory board and steering committee speaker roles from AstraZeneca, Bayer, and Boehringer Ingelheim/Lilly; participation as a board member of NKF of Southern California and Nevada and Wearable Artificial Kidney; and participation in the editorial board of the *American Journal of Nephrology*; is the Associate Editor for the *Journal of the American Society of Nephrology*; and reports honoraria from ASN Hypertension Highlights, ADA, and ASN nephSAP. Dr Neumiller reports personal fees and other support from Bayer AG, Boehringer Ingelheim, Dexcom, Eli Lilly, Novo Nordisk, Proteomics International, and Sanofi. Dr Mathew is an employee of the VHA; reports participation in the data safety monitoring committee for Procyrion-sponsored device trial and through central trial organizing committee Advarra; and reports equity ownership in Lowes, Apple, Snowflake, and Corning. Dr Gee reports self-employment at iAdvocate Inc, a health and wellness organization, and P. Gee Consulting, LLC; consultancy for FOUNTAIN EAC (Bayer International); Honoraria from American Kidney Fund, Amgen, APOLLO APOL1 Long-term Kidney Transplantation Community Advisory Council (CAC), Bayer International, Boehringer Ingelheim, CareDX, FOUNTAIN (Bayer International), NephCure International, Patient Family Advisors Network (PFA Network), Patient Family Center Care Partners (PFPC partners), Robert Woods Johnson Foundation, Traverse, and Vertex International; advisory or leadership roles for KHI Patient Family Partnership Council Chair, KHI Strategy BOD Member, University of Washington Center for Dialysis Innovation (CDI) Patient Advisory Board and Organ Procurement Transplant Network Kidney Transplantation Committee member, National Kidney Foundation Health Equity Advisory Board, National Kidney Foundation Kidney Advisory Council DEI Advisory Board, FOUNTAIN Executive Advisory Committee (Bayer International), and AHA Cardio-Kidney Metabolic Health Patient Advisory Group; Speakers bureau for CareDX; and other interests or relationships as AAKP Ambassador, AKF Ambassador and Kidney Health Coach, NKF KAC, UNOS Ambassador, PCORI Ambassador, NCC PFE-LAN SME, KHI PFPC Member, KPAC Member, Quality Insights Renal Network 5 PAC Chair, ASN Diabetic Kidney Disease Collaborative Task Force, CareDX Ambassador, *Kidney 360* Patient Perspective Ambassador, End Stage Renal Disease Health Equity Advisory Board, National Kidney Foundation Spring Clinical Meeting Planning Committee, and KHI APOL-1 Steering Committee. Dr Tuttle reports NIH research grants (U2CDK114886, UL1TR002319, U54DK083912, U01DK100846, OT2HL161847, UM1AI109568, and R01MD014712) and CDC project (number 75D301-21-P-12254); investigator-initiated grant support from Bayer and Travere outside of the submitted work; consultancy fees from AstraZeneca, Boehringer Ingelheim, Eli Lilly and Company, Novo Nordisk, and Travere; and speaker fees from AstraZeneca, Eli Lilly, and Novo Nordisk.
