# Supplementary material for: A Systematic Literature Review of Coordinated Care in Cardiovascular-Kidney-Metabolic Conditions
Source: Mayo Clin Proc Innov Qual Outcomes. 2025 Nov 12;9(6):100671. doi: 10.1016/j.mayocpiqo.2025.100671 (PMC12657295; doi:10.1016/j.mayocpiqo.2025.100671)
Supplement: Supplementary Material [file mmc1.docx]

**Supplementary materials**

# Supplementary Figure 1. Benefits of coordinated care programs for CKMs


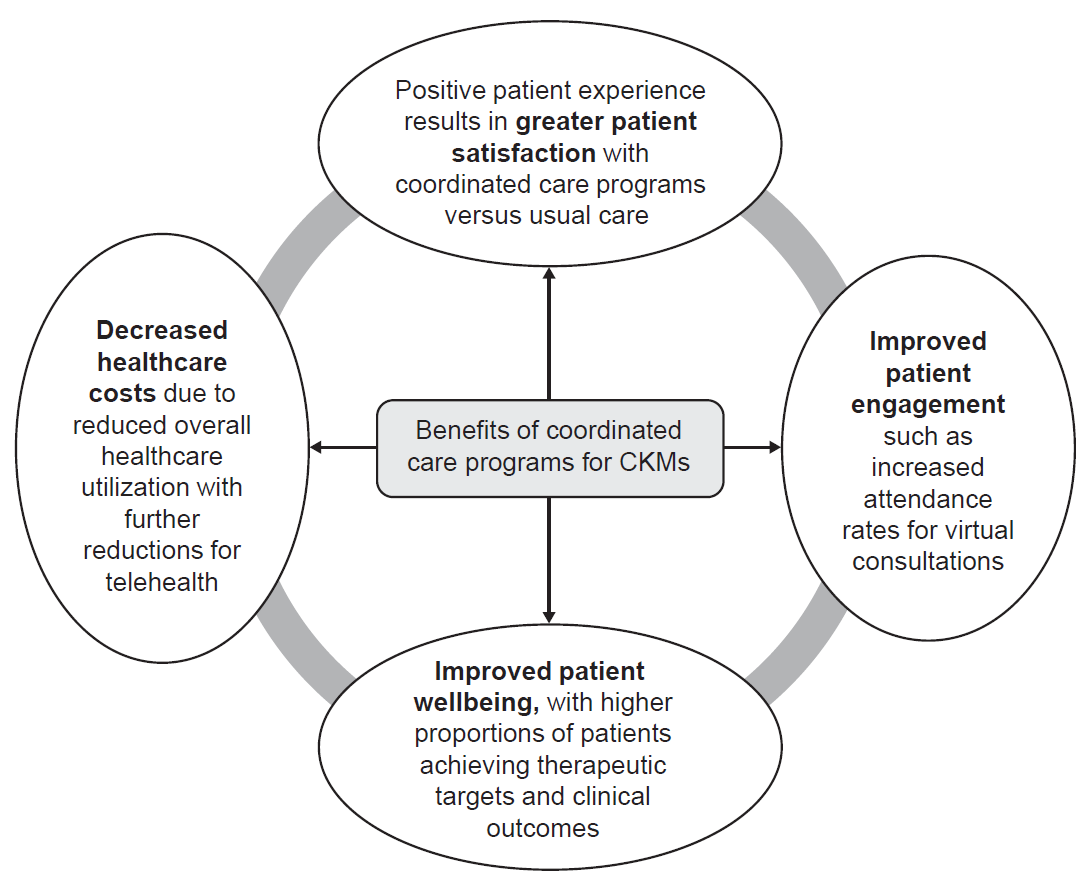


CKM, cardiovascular kidney metabolic.

# Supplementary Table 1a. Embase search strategy

Embase <1974 to 2023 March 09>

| **#** | **Query** | **Results from Mar 10, 2023** |
| --- | --- | --- |
| 1 | exp chronic kidney failure/ or chronic kidney disease/ or Diabetic Nephropathies/ or (chronic kidney or chronic renal or CKF or CKD or CRF or CRD or DKD or DRD or nephro$ or nephrit$ or glomerulo$ or renal or kidney).ti,ab,kw. | 1,520,127 |
| 2 | ((renal or kidney*) adj3 (insufficienc* or disease* or function* or failure* or dysfunction*)).ti,ab,kw. | 587,224 |
| 3 | 1 or 2 | 1,520,907 |
| 4 | non insulin dependent diabetes mellitus/ or diabet*.mp. or (NIDDM or T2D* or non insulin* depend* or noninsulin* depend* or noninsulin?depend* or non insulin?depend* or dietici*).ti,ab,kw. | 1,423,631 |
| 5 | ((typ? 2 or typ? II or typ?2 or typ?II) adj3 diabet*).ti,ab,kw. | 287,359 |
| 6 | metabolic syndrome X/ | 100,307 |
| 7 | (metabolic adj3 syndrome$1).mp. or (insulin adj3 resistan:).ti,ab,kw. | 243,494 |
| 8 | exp obesity/ or exp overweight/ | 644,380 |
| 9 | (obesity or obese).ti,ab,kw. | 563,913 |
| 10 | (overweight or over-weight or over weight or overeating or over eating or over-eating).ti,ab,kw. | 136,579 |
| 11 | or/4-10 | 1,976,942 |
| 12 | cardiovascular diseases/ or (Cardiovascular or heart or cardia* or coronar* or CVD or CVR or heart failure or stroke* or myocardial infarct* or MI or MACE or CV or transient isch?emi* or cardiac failure or CVOT or atheroscrelot* or atherosclerosis or dyslipidemia* or hypertension or elevated blood pressure or high blood pressure).ti,ab,kw. | 3,737,472 |
| 13 | ((risk or score or calcul*) adj5 (heart* or cardio* or cardia* or isch$em* or angina or coronary or infarct* or cvd or stroke or strokes)).ti,ab,kw. | 509,359 |
| 14 | or/12-13 | 3,754,147 |
| 15 | 3 and 11 and 14 | 117,612 |
| 16 | (cardio-renal-metaboli* or cardio-nephro-metaboli* or CVRM or cardiorenal metaboli* or cardiovascular-renal-metaboli* or cardiovascular-nephro-metaboli* or cardio-kidney-metaboli* or cardiovascular-kidney-metaboli* or cardiokidney metaboli*).mp. | 243 |
| 17 | 15 or 16 | 117,746 |
| 18 | exp integrated health care system/ or integrated care.mp. | 20,621 |
| 19 | (integrated health* and (organization* or organisation* or administration or insurance* or insurer* or provider*)).mp. | 10,609 |
| 20 | (multidisciplin* or multi-disciplin* or mdt or multispecial* or multi-special* or multipartner* or multi-partner* or multisector or multi-sector or multi-agency or multiagency or multiprofession* or multi-profession* or intraprofession* or intra-profession* or interprofession* or inter-profession* or transdisciplin* or trans-disciplin* or interdisciplin* or inter-disciplin* or intradisciplin* or intra-disciplin*).mp. | 323,526 |
| 21 | exp interdisciplinary communication/ | 13,439 |
| 22 | ((holistic or interdisciplinary or multidisciplinary or collaborat* or cooperati* or coordinat*) and care).mp. | 353,625 |
| 23 | ((interprofession* or inter-profession* or multidisciplin* or interdisciplin* or multispecial*) adj2 (collaborat* or team* or communicati* or network* or team* or staff* or meeting* or manag* or appointment* or intervention* or service* or approach* or system* or practice* or program* or advis* or advice* or caring or care or intervention* or communicat* or relation* or relate* or collaborat* or strateg* or model* or coordinat*)).mp. | 180,410 |
| 24 | (integrated service network or clinical integration or ((doctor* or physician* or pharmac* or nurse* or dietici* or nutrition*) adj5 collaborat$)).mp. | 15,371 |
| 25 | (Interprofessional Relations and (collaborat* or team* or coordinat*)).mp. | 405 |
| 26 | clinical pathway/ or guideline directed therapy.mp. | 9927 |
| 27 | ((healthcare or care) adj2 team*).ti,ab. | 37,921 |
| 28 | or/18-27 | 582,593 |
| 29 | 17 and 28 | 2596 |
| 30 | (conference abstract or editorial or comment* or letter or note or case study or case studies or case reports or erratum).pt. or (congress or case stud* or case report*).ti,ab,sh. | 10,443,188 |
| 31 | ((animal$ not human$) or nonhuman or human cell).sh,hw. or exp preclinical study/ | 10,504,720 |
| 32 | (in vitro or rat or rats or rodent or rodents or mouse or mice or murine or dog or dogs or canine or pig or pigs or porcine).ti,ab. | 5,226,827 |
| 33 | or/30-32 | 20,395,467 |
| 34 | 29 not 33 | 1357 |
| 35 | limit 34 to english language | 1205 |
| 36 | limit 35 to yr="2015 -Current" | 798 |

# Supplementary Table 1b. Embase (conference abstract) search strategy

Embase <1974 to 2023 March 09>

| **#** | **Query** | **Results from Mar 10, 2023** |
| --- | --- | --- |
| 1 | exp chronic kidney failure/ or chronic kidney disease/ or Diabetic Nephropathies/ or (chronic kidney or chronic renal or CKF or CKD or CRF or CRD or DKD or DRD or nglish$ or nephrit$ or glomerulo$ or renal or kidney).ti,ab,kw. | 1,520,127 |
| 2 | ((renal or kidney*) adj3 (insufficienc* or disease* or function* or failure* or dysfunction*)).ti,ab,kw. | 587,224 |
| 3 | 1 or 2 | 1,520,907 |
| 4 | non insulin dependent diabetes mellitus/ or diabet*.mp. or (NIDDM or T2D* or non insulin* depend* or noninsulin* depend* or noninsulin?depend* or non insulin?depend* or dietici*).ti,ab,kw. | 1,423,631 |
| 5 | ((typ? 2 or typ? II or typ?2 or typ?II) adj3 diabet*).ti,ab,kw. | 287,359 |
| 6 | metabolic syndrome X/ | 100,307 |
| 7 | (metabolic adj3 syndrome$1).mp. or (insulin adj3 resistan:).ti,ab,kw. | 243,494 |
| 8 | exp obesity/ or exp overweight/ | 644,380 |
| 9 | (obesity or obese).ti,ab,kw. | 563,913 |
| 10 | (overweight or over-weight or over weight or overeating or over eating or over-eating).ti,ab,kw. | 136,579 |
| 11 | or/4-10 | 1,976,942 |
| 12 | cardiovascular diseases/ or (Cardiovascular or heart or cardia* or coronar* or CVD or CVR or heart failure or stroke* or myocardial infarct* or MI or MACE or CV or transient isch?emi* or cardiac failure or CVOT or atheroscrelot* or atherosclerosis or dyslipidemia* or hypertension or elevated blood pressure or high blood pressure).ti,ab,kw. | 3,737,472 |
| 13 | ((risk or score or calcul*) adj5 (heart* or cardio* or cardia* or isch$em* or angina or coronary or infarct* or cvd or stroke or strokes)).ti,ab,kw. | 509,359 |
| 14 | or/12-13 | 3,754,147 |
| 15 | 3 and 11 and 14 | 117,612 |
| 16 | (cardio-renal-metaboli* or cardio-nephro-metaboli* or CVRM or cardiorenal metaboli* or cardiovascular-renal-metaboli* or cardiovascular-nephro-metaboli* or cardio-kidney-metaboli* or cardiovascular-kidney-metaboli* or cardiokidney metaboli*).mp. | 243 |
| 17 | 15 or 16 | 117,746 |
| 18 | exp integrated health care system/ or integrated care.mp. | 20,621 |
| 19 | (integrated health* and (organization* or organisation* or administration or insurance* or insurer* or provider*)).mp. | 10,609 |
| 20 | (multidisciplin* or multi-disciplin* or mdt or multispecial* or multi-special* or multipartner* or multi-partner* or multisector or multi-sector or multi-agency or multiagency or multiprofession* or multi-profession* or intraprofession* or intra-profession* or interprofession* or inter-profession* or transdisciplin* or trans-disciplin* or interdisciplin* or inter-disciplin* or intradisciplin* or intra-disciplin*).mp. | 323,526 |
| 21 | exp interdisciplinary communication/ | 13,439 |
| 22 | ((holistic or interdisciplinary or multidisciplinary or collaborat* or nglisha* or coordinat*) and care).mp. | 353,625 |
| 23 | ((interprofession* or inter-profession* or multidisciplin* or interdisciplin* or multispecial*) adj2 (collaborat* or team* or communicati* or network* or team* or staff* or meeting* or manag* or appointment* or intervention* or service* or approach* or system* or practice* or program* or advis* or advice* or caring or care or intervention* or communicat* or relation* or relate* or collaborat* or strateg* or model* or coordinat*)).mp. | 180,410 |
| 24 | (integrated service network or clinical integration or ((doctor* or physician* or pharmac* or nurse* or dietici* or nutrition*) adj5 collaborat$)).mp. | 15,371 |
| 25 | (Interprofessional Relations and (collaborat* or team* or coordinat*)).mp. | 405 |
| 26 | clinical pathway/ or guideline directed therapy.mp. | 9927 |
| 27 | ((healthcare or care) adj2 team*).ti,ab. | 37,921 |
| 28 | or/18-27 | 582,593 |
| 29 | 17 and 28 | 2596 |
| 30 | (editorial or comment* or letter or note or case study or case studies or case reports or erratum).pt. or (case stud* or case report*).ti,ab,sh. | 6,083,350 |
| 31 | ((animal$ not human$) or nonhuman or human cell).sh,hw. Or exp preclinical study/ | 10,504,720 |
| 32 | (in vitro or rat or rats or rodent or rodents or mouse or mice or murine or dog or dogs or canine or pig or pigs or porcine).ti,ab. | 5,226,827 |
| 33 | or/30-32 | 17,142,979 |
| 34 | 29 not 33 | 2168 |
| 35 | conference abstract.pt. | 4,694,033 |
| 36 | 34 and 35 | 808 |
| 37 | limit 36 to english language | 808 |
| 38 | limit 37 to yr=”2021 -Current” | 154 |

# Supplementary Table 1c. Medline search strategy

Ovid MEDLINE(R) and Epub Ahead of Print, In-Process, In-Data-Review & Other Non-Indexed Citations, Daily and Versions <1946 to March 09, 2023>

| **#** | **Query** | **Results from Mar 10, 2023** |
| --- | --- | --- |
| 1 | Renal Insufficiency/ or Kidney Failure/ or exp Renal Insufficiency, Chronic/ or Kidney Diseases/ or Diabetic Nephropathies/ or (chronic kidney or chronic renal or CKF or CKD or CRF or CRD or DKD or DRD or nephro$ or nephrit$ or glomerulo$ or renal or kidney).ti,ab,kw. | 1,120,350 |
| 2 | ((renal or kidney*) adj3 (insufficienc* or disease* or function* or failure* or dysfunction*)).ti,ab,kw. | 383,722 |
| 3 | 1 or 2 | 1,121,068 |
| 4 | Diabetes Mellitus, Type 2/ or diabet*.mp. or (NIDDM or T2D* or non insulin* depend* or noninsulin* depend* or noninsulin?depend* or non insulin?depend*).ti,ab,kw. | 826,165 |
| 5 | ((typ? 2 or typ? II or typ?2 or typ?II) adj3 diabet*).ti,ab,kw. | 181,887 |
| 6 | metabolic syndrome/ | 37,704 |
| 7 | (metabolic adj3 syndrome$1).mp. or (insulin adj3 resistan:).ti,ab,kw. | 152,652 |
| 8 | exp obesity/ or exp overweight/ | 266,973 |
| 9 | (obesity or obese).ti,ab,kw. | 372,471 |
| 10 | (overweight or over-weight or over weight or overeating or over eating or over-eating).ti,ab,kw. | 89,509 |
| 11 | or/4-10 | 1,200,049 |
| 12 | Cardiovascular diseases/ or (Cardiovascular or heart or cardia* or coronar* or CVD or CVR or heart failure or stroke* or myocardial infarct* or MI or MACE or CV or transient isch?emi* or cardiac failure or CVOT or atheroscrelot* or atherosclerosis or dyslipidemia* or hypertension or elevated blood pressure or high blood pressure).ti,ab,kw. | 2,647,830 |
| 13 | ((risk or score or calcul*) adj5 (heart* or cardio* or cardia* or isch$em* or angina or coronary or infarct* or cvd or stroke or strokes)).ti,ab,kw. | 315,604 |
| 14 | or/12-13 | 2,658,025 |
| 15 | 3 and 11 and 14 | 56,011 |
| 16 | (cardio-renal-metaboli* or cardio-nephro-metaboli* or CVRM or cardiorenal metaboli* or cardiovascular-renal-metaboli* or cardiovascular-nephro-metaboli* or cardio-kidney-metaboli* or cardiovascular-kidney-metaboli* or cardiokidney metaboli*).mp. | 160 |
| 17 | 15 or 16 | 56,103 |
| 18 | "delivery of health care"/ or "delivery of health care, integrated"/ | 128,179 |
| 19 | exp Interprofessional Relations/ and (collaborat$ or team$).tw. | 17,081 |
| 20 | (integrated health* and (organization* or organisation* or administration or insurance* or insurer* or provider*)).mp. | 3146 |
| 21 | (multidisciplinary or multi-disciplinary or multispecial* or multi-special* or mdt or multipartner* or multi-partner* or multisector or multi-sector or multi-agency or multiagency or multiprofessional or multi-professional or intraprofessional or intra-professional or interprofessional or inter-professional or transdisciplinary or trans-disciplinary or interdisciplinary or inter-disciplinary or intradisciplinary or intra-disciplinary).tw. | 189,965 |
| 22 | interdisciplinary communication/ | 18,127 |
| 23 | ((holistic or interdisciplinary or multidisciplinary or cooperati* or collaborati* or coordinat*) and care).mp. | 203,499 |
| 24 | ((interprofession* or inter-profession* or multidisciplin* or interdisciplin* or multispecial*) adj2 (collaborat* or team* or communicati* or network* or team* or staff* or meeting* or manag* or appointment* or intervention* or service* or approach* or system* or practice* or program* or advis* or advice* or caring or care or intervention* or communicat* or relation* or relate* or collaborat* or strateg* or model* or coordinat*)).mp. | 163,291 |
| 25 | (integrated service network or clinical integration or ((doctor* or physician* or pharmac* or nurse* or dietici* or nutrition*) adj5 collaborat$)).mp. | 10,045 |
| 26 | coordination of care.mp. | 2284 |
| 27 | ((healthcare or care) adj2 team*).ti,ab. | 23,636 |
| 28 | patient care planning/ or clinical pathway.mp. or guideline directed therapy.mp. | 42,181 |
| 29 | or/18-28 | 527,203 |
| 30 | 17 and 29 | 1168 |
| 31 | (editorial or comment* or letter or note or case study or case studies or case reports).pt. or (case stud* or case report* or congress).ti,ab,sh. | 4,510,182 |
| 32 | ((animals not (humans and animals)) or in vitro or nonhuman).sh. or (in vitro or rat or rats or rodent or rodents or mouse or mice or murine or dog or dogs or canine or pig or pigs or porcine or bovine or cow).ti,ab. | 6,879,061 |
| 33 | or/31-32 | 11,226,467 |
| 34 | 30 not 33 | 1083 |
| 35 | limit 34 to english language | 967 |
| 36 | limit 35 to yr="2015 -Current" | 601 |

# Supplementary Table 2a. NOS quality assessment

| **Author, year** | **Selection** | | | | **Comparability** | **Outcome** | | | **Total** |
| --- | --- | --- | --- | --- | --- | --- | --- | --- | --- |
|  | **Representativeness of the exposed cohort** | **Selection of the non exposed cohort** | **Ascertainment of exposure** | **Demonstration that outcome of interest was not present at start of study** | **Comparability of cohorts on the basis of the design or analysis** | **Assessment of outcome** | **Was follow-up long enough for outcomes to occur** | **Adequacy of follow up of cohorts** |  |
| Burke 2023^20^ | 1 | 1 | 1 | 1 | 2 | 1 | 0 | 0 | 7 |
| Dubrofsky 2022^4^ | 1 | 0 | 1 | 1 | 0 | 1 | 1 | 1 | 6 |
| Essa 2022^24^ | 1 | 1 | 1 | 1 | 0 | 1 | 1 | 1 | 7 |
| Katz 2018^21^ | 1 | 1 | 1 | 1 | 0 | 1 | 1 | 1 | 7 |
| Li 2021^8^ | 1 | 0 | 1 | 1 | 0 | 1 | 1 | 0 | 5 |
| Lim 2020^9^ | 1 | 1 | 1 | 1 | 2 | 1 | 1 | 1 | 9 |
| Lu 2021^22^ | 1 | 1 | 1 | 1 | 2 | 1 | 1 | 1 | 9 |
| Neeland 2022^11^ | 1 | 1 | 1 | 1 | 0 | 1 | 1 | 1 | 7 |
| Schutze 2021^12^ | 1 | 1 | 1 | 1 | 0 | 1 | 1 | 1 | 7 |
| Triantafylidis 2021^14^ | 1 | 0 | 1 | 1 | 0 | 1 | 0 | 1 | 5 |
| Vu 2022^19^ | 1 | 0 | 1 | 1 | 0 | 1 | 0 | 1 | 5 |

NOS, Newcastle-Ottawa Scale.

# Supplementary Table 2b. Quality scoring of RCTs based on RoB2

| **Author / trial ID** | **Trial name** | **Study design** | **Description/ Judgement** | **Was the allocation sequence random?** | **Was the allocation sequence concealed until participants were enrolled and assigned to interventions?** | **Was knowledge of the allocated interventions adequately prevented from participants and personnel** | **Was knowledge of the allocated interventions adequately prevented from outcome assessors** | **Were incomplete outcome data adequately addressed?** | **Are reports of the study free of suggestion of selective outcome reporting?** | **Was the study apparently free of other problems that could put it at a high risk of bias?** |
| --- | --- | --- | --- | --- | --- | --- | --- | --- | --- | --- |
| Anderegg 2018^16^ | CAPTION trial (NCT00935077) | Post-hoc analysis of RCT | Judgement | Yes | Yes | No | No | Yes | Yes | Yes |
| Anderegg 2018^16^ | CAPTION trial (NCT00935077) | Post-hoc analysis of RCT | description | Offices were randomized to one of the three study arms: (i) usual BP care; (ii) a 9-month BP intervention; or (iii) a sustained, 24-month intervention. The two intervention arms were designed to be the same for the first 9 months so they could be combined and compared to usual care. | Patients were identified using billing records to identify those with hypertension. Patients lists were then submitted to a biostatistician for randomization | Open Label study | Open Label study | Blood pressure was assumed to be uncontrolled if a BP value was missing at 9 months - a conservative method to recognize that if a patient missed the visit, BP was more likely to be uncontrolled than controlled. | All outcomes specified in the method were reported. | A study coordinator used billing records to identify patients with hypertension. Biostatistician randomized the patients. The study coordinator then selected patients in order from the randomized list to avoid selection bias |
| Dixon 2021^17^ | CAPTION trial (NCT00935077) | Post-hoc analysis of RCT | Judgement | Yes | Yes | No | No | Unclear | Yes | Yes |
| Dixon 2021^17^ | CAPTION trial (NCT00935077) | Post-hoc analysis of RCT | description | Offices were randomized to one of the three study arms: (i) usual BP care; (ii) a 9-month BP intervention; or (iii) a sustained, 24-month intervention. The two intervention arms were designed to be the same for the first 9 months so they could be combined and compared to usual care. | Patients were identified using billing records to identify patients with hypertension. Patients’ lists were then submitted to a biostatistician for randomization | Open Label study | Open Label study | Text on page 6 reports missing systolic BP data, but does not say how it was accounted for in the analysis | All outcomes specified in the method were reported. | None identified |
| Chan 2022^3^ | A JADE RCT (NCT02176278) | RCT | Judgement | Yes | Yes | No | No | Yes | Yes | Unclear |
| Chan 2022^3^ | A JADE RCT (NCT02176278) | RCT | description | Patients were randomized in a 1:1:1 ratio at each site to usual care, empowered care, or team-based empowered care. | Computer-generated assignment codes were put in sealed, opaque, and consecutively numbered envelopes and then opened by non–study personnel at the site. | Open Label study - Patients, investigators, and nurses were not blinded according to the design of the study. | Open Label study - Patients, investigators, and nurses were not blinded according to the design of the study. | Missing data were handled by multiple imputation by chained equations with 20 imputations. | All outcomes specified in the methods were reported. | 159 patients in the empowered care group and 192 patients in the team-based empowered care group did not adhere to protocol and were excluded as opposed to only 4 patients in the usual care group. Characteristics of these patients may have introduced a bias in the study results. |
| Keel 2020^6^ | CareHND (NCT03362983) | RCT | Judgement | Yes | Unclear | No | No | Unclear | Yes | Yes |
| Keel 2020^6^ | CareHND (NCT03362983) | RCT | Description | Inclusion and exclusion criteria were defined according to the ongoing randomized control trial | No details provided in protocol (Spaak et al 2015); this is a secondary cost-analysis | No masking. As the intervention was enrollment in a multidisciplinary center (HND center) vs usual care, the participants would have been aware. | No masking was done. It was an open-label trial. | No data provided [including no information in protocol]; this is a cost analysis (time-driven activity-based cost [TDABC]) | All outcomes specified in the method were reported | None identified |
| Rafiq 2019^7^ | CareHND (NCT03362983) | RCT | Judgement | Yes | Unclear | No | No | Yes | Yes | Yes |
| Rafiq 2019^7^ | CareHND (NCT03362983) | RCT | Description | Patients were randomized to either traditional standard care or to the HND center. | No details provided in the protocol (Spaak et al 2015) | No masking. The intervention was enrollment in a multidisciplinary program. Patients were aware, as they were attending appointments. | No masking was done. It was an open-label trial. | Data were cleaned by eliminating missing values. After removing missing values, the data was normalized to a common time scale. | All outcomes specified in the method/protocol were reported. | None identified |
| Lear 2021^18^ | iCDM (NCT01342263) | RCT | Judgement | Yes | Yes | No | Yes | Partial yes | Yes | Yes |
| Lear 2021^18^ | iCDM (NCT01342263) | RCT | Description | Participants were randomized on a 1:1 ratio to receive either usual care or the internet CDM program using variable block sizes | A randomization assistant conducted the randomization after patients had been enrolled and patients were then informed of their group assignment. | The randomization assistant informed participants of their group assignment | Group assignment was not revealed to the study research coordinators to retain study blinding | Sensitivity analysis was conducted to exclude patients who died during the study. No additional info. | All outcomes specified in the method were reported. | None identified |

BP, blood pressure; CAPTION, Collaboration Among Pharmacists and Physicians to Improve Outcomes Now; CareHND, Care Heart Nephrology Diabetes; iCDM, Internet-based Platform for Chronic Diseases Management; JADE, Joint Asia Diabetes Evaluation; RCT, randomized controlled trial; RoB2, Cochrane risk of bias tool, version 2.

# Supplementary Table 3. Interventions, grouped by setting (n=22)

| **Author, year (country)** | **Intervention/study name (NCT) & type** | **Provider types Intervention led by?** | **Program objectives** | **Program summary** | **Type of patient interaction** | **Frequency of assessment** | **Resources required** | **Program-related challenges** |  |
| --- | --- | --- | --- | --- | --- | --- | --- | --- | --- |
| **Outpatient specialist clinic (n=14 interventions in 15 publications)** | | | | | | | | | |
| Al-Chalabi 2022 (UK) [Congress abstract]^1^ | MRC clinic  Type: Patient visits | MST: Nephrologists, diabetology and cardiology input (providers NR)  Nephrologist-led | To provide diabetology and cardiology input in addition to nephrology care for patients with cardio-renal syndrome | NR | In-person clinic visits | NR | NR | NR; occurred during COVID-19 |  |
| Comaschi 2020 (Italy) [Protocol]^2^ | INSIDE study  Type: Patient visits | MDT & MST: Physicians (including GP and cardiologist), nurses, dietician, podologist, diabetes specialist, social workers  GP-led | To create a national integrated care organization comprising primary care, diabetes care, and CVD care to improve treatment adherence and achieve guideline targets in patients with T2DM at high risk for CVD | Integrated care organization involving 3 different levels of care. Patients to be enrolled by primary care centers; GP will conduct initial tests. Diabetes specialist will conduct educational training and medication changes. Cardiology to be consulted based on test results | In-person clinic visits | Follow-up visits: 180, 360, and 720 days | Multidisciplinary and interdisciplinary care providers; access to certified labs, imaging (eg, doppler scans, CT), ECG machine; Electronic case report form | Intensive patient education sessions – therefore likely to only be done in diabetes centers. Use of several different laboratories for examination measurements, no centralization |  |
| Chan 2022 (Asia*)^3^ | A JADE RCT (NCT02176278)  Type: Patient engagement/  education | MDT: Physicians (type NR), nurses  Nurse-led | To empower self-management/care tailored to the needs of patients with DKD | Patients were recruited/screened using JADE web platform and were randomized to 1 of 3 care groups (**usual care, empowered care, or team-based empowered care**). All 3 groups received standard care; but those randomized to empowered or team-based empowered care received additional healthcare services and follow-up | In-person and telephone | All 3 groups were assessed at baseline and after 12 months **Usual care:** follow-up according to practice at the site **Empowered:** follow-up according to practice at the site + follow-up by nurse every 3 months **Team-based empowered:** same as empowered care group + clinic visit every 3 months, managed by team of 1 doctor and 1 nurse | Multidisciplinary care providers, site investigators, statisticians, technology devices, telephone, ICT, laboratories (for tests). Each participating center received a grant equivalent to an 18-month nurse salary | 2 sites did not enroll patients due to administrative delay |  |
| Dubrofsky 2022 (Canada)^4^ | C.a.R.E. Clinic  Type: Patient visits | MDT & MST: Nephrologist, cardiologist, endocrinologist, pharmacist, dietician, chiropodist, ophthalmologist, clinical fellow, diabetes nurse educator  Program lead: NR | To provide multi- and interdisciplinary care to patients with diabetes and co-existing CKD and/or CVD to overcome barriers to optimal care | Patients may be seen by multiple providers on a single appointment day.  The multi- and interdisciplinary care team review treatment plans for each patient to ensure consistent messaging and care plans | In-person clinic visits | Initial visit + as needed. Clinic held once monthly | Multidisciplinary and interdisciplinary care providers; access to hospital labs, imaging, and resources | Non-attendance rate was 17–18%. BMI did not improve between first and last clinic visit. No data on medication adherence or health behavior changes, therefore difficult to ascertain what component of the intervention was most effective |  |
| Jegatheesan 2022 (Australia) [Congress abstract]^5^ | LANDMARK III (multidisciplinary lifestyle intervention)  Type: Patient engagement/ education Patient visits | MDT & MST: Nurse, exercise physiologist, dietitian, psychologist, diabetes educator, and social worker  Nurse-led | To assess the effect of a nurse-led model of care on CV risk factor modification in patients with moderate CKD | Multidisciplinary intervention consisting of lifestyle and medication advice for risk factor management, an exercise training program, and a behavioral modification program | Unclear; appears to be in-person clinic visits | Transthoracic echocardiogram: Baseline and annually | Multidisciplinary care providers (for 8-wk exercise training program, 4-wk behavioral modification program), other resources NR | NR |  |
| Keel 2020 (Sweden)^6^ | CareHND (NCT03362983)  Type: Patient visits | MDT & MST: Registered nurses, doctors, health care assistants/ 'underskoterska'  Program lead: NR | [Spaak 2015 (protocol-supporting material): To provide a novel integrated outpatient clinic, resulting in better care and lower overall burden on the healthcare system] | A multidisciplinary and integrated care delivery center focused on providing care for patients with HND conditions | In-person clinic visits, telephone consultation | NR *[See Rafiq 2019]* | Multidisciplinary and interdisciplinary care providers; telephone equipment; clinical chemistry labs, imaging, and resources | Costing methodology required adaptations for this particular patient group, stratifying costs by activity and resource |  |
| Rafiq 2019 (Sweden)^7^ | CareHND (NCT03362983) Type: Patient visits | MDT & MST: HND staff: Junior and senior consultants, nurse managers, healthcare assistants/underskoterska (equivalent of an American Licensed Practical Nurse or a British NHS Health Care Assistant); plus dieticians, physiotherapists, and nurses specialized in HF, nephrology, and diabetes; also with nephrologists, cardiologists, and endocrinologists  Program lead: NR | To offer comprehensive, integrated, multidisciplinary, and person-centered care to HND patients to improve care coordination, reduce unnecessary health care utilization, lower costs, and within a 1-year period, develop a sustainable care management plan that can be handed-off to primary care | Multidisciplinary center where patients meet the team through outpatient visits, and are discussed in regular internal team meetings. Instead of seeking treatment at different locations by specialty, patients obtain all treatments at this single location | In-person clinic visits, telephone calls | Patients typically receive 3–4 outpatient visits, 1 inpatient visit, and 3–5 days in hospital per admission; Junior consultant discusses patients with 3 specialists at bi-weekly conferences | Multidisciplinary and interdisciplinary care providers; telephone equipment | Delivering care to treat 59 separate diagnoses was a challenge for the providers and researchers |  |
| Li 2021 (USA)^8^ | Pharmacist–physician collaborative care  Type: pharmacist integration | MDT: Pharmacist, nephrologist  Pharmacist-led | To incorporate pharmacist-led medication management with partnering nephrologists while capitalizing on technology to improve outcomes in a CKD population | Nephrologist–pharmacist partnership to incorporate pharmacist-driven medication management while utilizing technology (remote BP monitoring in patients with uncontrolled HTN) to improve outcomes in a CKD population | In-person visits initially, but telehealth during and after COVID-19 pandemic | NR | Blood pressure monitor kit for each HTN patient; computer tablet, technology access (devices, internet, electronic medical record system, etc.), home logbook for patient | NR |  |
| Lim 2020 (Hong Kong)^9^ | Technology-assisted integrated care  Type: Patient engagement/education | MDT: Nurses, doctors, endocrinologists, internists, and primary care physicians  Nurse-led | To advance the use of diabetes centers, ICT, and nonphysician personnel to evaluate, empower, and engage patients with T2DM to promote self-management that complements physician care | Nurse-led structured evaluation (blood/urine/eye/feet) in public and private outpatient clinics and diabetes centers in Hong Kong; **JADE group** also received JADE report + nurse-led group empowerment; **JADE-P group** also received JADE report (patients paid $300) + nurse-led personal empowerment + annual telephone reminder for re-evaluation | In-person (+ telephone reminders for JADE-P) | NR (JADE-P cohort received annual telephone reminder) | Diabetes centers, ICT, hospital resources, primary care clinics, technology devices, internet access, IT support (for JADE platform) **JADE-P**: patients required to pay a fee ($300) | NR |  |
| Narain 2022 (UK) [congress abstract]^10^ | CMC  Type: Patient visits | MST: Cardiologist, diabetologist  Program lead: NR | To facilitate medication optimization and lifestyle interventions to reduce cardiometabolic risk | Cardiometabolic clinic facilitating medicines optimization and lifestyle intervention, where patients are seen by multidisciplinary team | Virtual (due to COVID-19) | NR | Multidisciplinary care providers; clinical labs for testing | Availability of clinical outcomes is limited due to the short period of follow-up so far. |  |
| Neeland 2022 (USA)^11^ | CINEMA program  Type: Patient visits | MDT & MST: Program administrator, cardiologist, nurse coordinator, certified diabetes care and education specialist who is also a registered dietitian, nutritionist, endocrinology, and nephrology providers  Program lead: NR (primarily managed by nurses) | To improve CV risk factors and increase use of evidence-based therapies to improve care for patients with T2DM | CINEMA is structured around 2–3 primary visits with follow-up visits as required to address all aspects of T2DM and CVD care. The care team comes to the patient (in-person or virtual) and completes assessment during initial visit. Patients receive continued support via telephone or virtual meetings. Patients return after 3 months for tests and to check progress. Patients continue routine follow-up with primary care and specialty physicians, and CINEMA physicians/support staff | In-person, telephone, virtual | Multiple visits/virtual meetings (initial, 3 months, weekly via podcast, routine primary/specialty care) | Multidisciplinary care providers, pharmacy, hospital resources, laboratories, single electronic health record system, educational resources, peer-led support groups, staff to contact patient insurance/medication assistance programs | Challenges getting “buy-in” from all stakeholders (eg, endocrinologists, nephrologists, certified diabetes care and education specialist, and business practice managers)  Concerns that specialist-driven care model may further fragment the role of PCPs in managing comorbid T2DM and CVD, could increase care costs, and worsen health disparities for patients unable to access specialist care |  |
| Schutze 2021 (Germany)^12^ | NR (adding clinical pharmacist to therapeutic team)  Type: pharmacist integration, GP training/education | MDT: Nephrologist, pharmacist, GP  Pharmacist-led | To improve medication optimization and safety in patients with CKD and increase GPs' acceptance of nephrologist's medication recommendations by involving a clinical pharmacist in the therapeutic team | Patients are assessed by the nephrologist and clinical pharmacist, who propose specialized medication recommendation to the GP. Clinical pharmacist conducts 3-month follow-up. At 6 months, GPs acceptance of treatment recommendations were assessed | In-clinic visits, telephone | Baseline,  3 months,  6 months | Multidisciplinary care providers; telephone consultation equipment | Relied on patient self-reports of medications taken and side effects; training courses could not be delivered to all GPs; unable to recruit the same number of patients in control/ intervention group due to administration changes |  |
| Tan 2019 (Singapore) [protocol]^13^ | IDEALS program (NCT03413215)  Type: Patient engagement/education | MDT & MST: Endocrinologist, diabetes nurse educator, a renal pharmacist, medical social workers, and dieticians  Program lead: NR | To assess whether an integrated program with patient empowerment would be more effective than usual care in controlling diabetes, CV risk factors, and nephropathy progression, in reducing clinical event rates, and in decreasing specialist clinic visits and hospitalizations | Patients will be managed by multiple providers trained in specific aspects of condition management. Endocrinologists will review test results and clinic measurements and adjust medication accordingly. Nurses will have frequent contact with patient and conduct clinical assessments for the physician. Renal pharmacists will optimize BP medication where required. Dieticians will counsel patients on nutrition. Medical social workers will focus on health behavior change | In-person clinic visit, telephone | NR [pharmacists: 2 weeks to 3 months; medical social workers: 3-weekly intervals] | Multidisciplinary care providers; BP monitors, glucometers, test strips and lancets; computer tablets; smartphone and/or notebooks (for logs); educational videos; software for femoral artery measurements; laboratories for testing; telephone for follow-ups | NR |  |
| Triantafylidis 2021 (USA)^14^ | NR (embedding clinical pharmacist within interprofessional nephrology clinic)  Type: Pharmacist integration | MDT & MST: Nephrologists and pharmacists. Pharmacist notified PCP and diabetes management team of medication changes via email.  Pharmacist-led | To use an interprofessional clinic model to optimize initiation and monitoring of SGLT2 inhibitors (empagliflozin) in patients with DKD | Clinical pharmacist was embedded within nephrology clinic to provide patient education, telephone follow-up, and collaborate with nephrologists for initiation and monitoring of empagliflozin, including adjusting diabetes, HTN, and diuretic regimens as needed | In-person clinic visits, telephone follow-up calls | Intensive pharmacist follow-up by telephone: week 2 and 6 and every 2–4 weeks thereafter as necessary. In-clinic follow-up every 3–6 months (or more often if needed) | Pharmacist and nephrologist. Access to labs and clinic; BP cuff and blood glucose testing supplies for self/home monitoring; telephone and email. For all pharmacist activities over the initial 3-month period, each patient required an average of 4.8 hours of pharmacist time | NR (but limited enrollment, n=14 patients) |  |
| Wongprasert 2022 (Thailand) [congress abstract]^15^ | MDT Care + Patient Empowerment (NCT: NR)  Type: Patient engagement/  education | MDT & MST: Endocrinologist, diabetes nurse educator, dietitian, renal pharmacist, and social worker for counseling  Program lead: NR | Intensive MDT care and patient empowerment | To empower patients with DKD to manage their care. Patients received care and education from MDT and were provided self-care empowerment tools | NR [appears to include in- person clinic visits; other interaction NR] | NR (probably monthly as visual abstract graph displays monthly % with good HbA1c control over 12 months) | Multidisciplinary care providers, case manager, hospital clinic facilities/labs, self-care tools for patients (glucometers, BP monitors, and calendars) | NR |  |
| **Primary care practices (n=3 interventions in 4 publications)** | | | | | | | | | |
| Anderegg 2018 (USA)^16^ | CAPTION trial (NCT00935077)  Type: pharmacist integration | MDT: Physician, Pharmacist  Pharmacist-led | As below (Dixon 2021) | As below (Dixon 2021) | As below (Dixon 2021) | As below (Dixon 2021) | BP measurement devices, multidisciplinary care providers, study coordinator, biostatistician, training facilities for study coordinator | NR |  |
| Dixon 2021 (USA)^17^ | CAPTION trial (NCT00935077)  Type: pharmacist integration | MDT: Physician, Pharmacist  Pharmacist-led | To improve SBP control/to achieve longer time in target range for SBP | Pharmacist devises care plan based on patient assessment; physician implements plan. Primary care practices were randomized to either a brief, 9 months or sustained, 24 months pharmacist intervention or  usual care (control group) | Telephone and face-to-face | At enrollment, 6, 9, 12, 18, and 24 months | BP measurement devices, multidisciplinary care providers | NR |  |
| Lear 2021 (Canada)^18^ | iCDM (NCT01342263)  Type: Patient engagement/  education Behavioral change | MDT & MST: Intervention: nurse with referrals for dietitian, exercise specialist, PCP  iCDM advisory committee: clinical researchers, PCPs, specialist physicians, allied health care professionals, digital health care professionals, and 3 patient members Nurse-led | To promote self-management and symptom monitoring in patients with ≥2 of the following 5 conditions: diabetes, HF, IHD, CKD, or COPD | Patients enrolled from primary care clinics in small urban and rural areas without ambulatory care clinics related to the target diseases.  iCDM group – health assessed at BL; patients provided login/training for CDM website. Patients prompted by email to complete symptom report daily for 2 wks, reduced to once per wk if no alerts. Alerts generated based on treatment algorithm; actions could include continued support with self-management, nurse contacting PCP for patient follow-up, or referral to nearest hospital. Every 8 wks, patients answered lifestyle questionnaire. Patients had access to public forum, their biometric data/alerts and action plan, and external online resources | Usual care: in-person (assumed) iCDM: web-based platform; telephone | Usual care: baseline, 12 months, 24 months  iCDM: BL, daily for 2 wks, 1 x weekly if no symptoms, lifestyle questionnaire every 8 wks | MDT team, study coordinator, telephone, internet, technology devices, staff to train patients on iCDM website, public forum, external online resources | NR but 70% of PCPs lacked electronic health record access at program start, so could not participate |  |
| Vu 2022 (USA)^19^ | Positive Kidney Health  Type: Pharmacist integration | MDT: Pharmacist, physician, education specialists, nephrologists Pharmacist-led | Patient education and risk factor control in patients with early CKD and those at risk for CKD | Pharmacists (embedded in primary care centers) were recruited and trained, who then worked in MDT to educate patients at risk for or with early CKD. Pharmacists had multiple in-person and video consultations with the patients to review risk factors and medications | In-person clinic visit or video visit (telehealth) | NR Total 47 visits (31 in-clinic, and 16 by video) during the pilot period  First visit; following visits at 2 or 4 weeks; no maximum number of follow-up visits | Multidisciplinary care providers; Educational workbook, CKD specific educational resource, Terasaki institute and UCLA facilities/resources, consulting services on graphic design and plain language editing, BP monitor and glucometer for home use, lifestyle counseling, behavioral treatment | Challenges to identify eligible patients (n=20); attending return visits to complete the 2- or 3-visit educational series was a challenge for some participants (research team often had to reschedule no-show visits) |  |
| **Primary/telemedicine with MDT (n=3 interventions in 3 publications)** | | | | | | | | | |
| Burke 2023 (USA)^20^ | NR (telemedicine between primary care site and multispecialty distance site)  Type: Patient visits | MDT & MST: Federally qualified health center: primary care provider, nurse/medical assistant;  distance site: endocrinologist, cardiologist, nephrologist  Program lead: NR | To determine if telemedicine consultation for specialist care reduces barriers associated with in-person visits in urban underserved patients, and to improve visit no-show rate, clinical outcomes, and patient satisfaction | Telemedicine consultation conducted from a dedicated exam room at the Federally qualified health center (originating site in primary care) and a specialist within an academic medical center (distance site; multispecialty group) | In-person visit (primary care), telemedicine (specialist care) | NR [telemedicine consultation; likely once] | Primary care and multispecialty care providers; telemedicine equipment; examination room and equipment | Access issues in reaching the primary care center for the physical examination |  |
| Katz 2018 (Australia)^21^ | iConnect CKD  Type: Patient visits | MDT & MST: Nephrologist, endocrinologist, cardiologist, renal palliative supportive care, and clinical nurse specialist (case manager).  Nurse and nephrologist-led | To provide a web-based virtual medical consult program to follow-up patients with CKD | Patients were stratified by risk status. Low-risk patients were monitored using a virtual consulting program to review patient records and devise treatment plan without patient being present; high-risk patients were randomized to either virtual consulting or face-to-face care | Virtual consultation (patients not present), in-person GP visits; patients followed up/monitored virtually | Follow-up for at least 6-monthly cycles | IT software; multidisciplinary care providers; remote monitoring equipment; telephone | System was old /hard to use; problems with IT integration; overall low enrollment from GPs; GPs wanted decision support and not to be involved in long-term follow-up; not all GPs and patients completed the qualitative surveys (13 GPs; 27 patients); patients were lost to follow-up or died |  |
| Lu 2021 (USA)^22^ | V-IMPACT  Type: Patient visits | MDT: Primary care providers, nurse coordinators, pharmacists, social workers, medical assistants, nurses, clerks  Program lead: NR | To utilize videoconferencing to connect primary care providers with rural veterans and increase their access to primary care | A hub-and-spoke model, where a hub team of professionals works with a local team to offer longitudinal, team-based primary care via video appointments | In-clinic (local team), appointment with remote MDT | NR | Multidisciplinary care providers; video conferencing software; digital stethoscopes and high-definition cameras; testing equipment | NR |  |
| **MDT meetings (n=2 interventions in 3 publications)** | | | | | | | | | |
| Essa 2021 (UK) [Congress abstract]^23^ | NR (HF multispecialty MDT virtual meetings)  Type: MDT meetings (virtual) | see below (Essa 2022) | see below (Essa 2022) | see below (Essa 2022) | see below (Essa 2022) | see below (Essa 2022) | Teleconference resources; multispecialty + multidisciplinary care providers. MDT meeting total costs (Jan–Dec 2020) were £15,400 and the resulting 31 clinic appointments cost ~£3720 | see below (Essa 2022) |  |
| Essa 2022 (UK)^24^ | NR (HF multispecialty MDT virtual meetings) Type: MDT meetings (virtual) | MDT & MST: HF cardiologists (primary, secondary, tertiary care), HF nurses, nephrologist, endocrinologist, palliative care specialist, chest physician, pharmacist, clinical pharmacologist, and geriatrician  Program lead: NR | To use a multispecialty approach to manage HF and comorbidities and improve outcomes; to integrate primary care community services with secondary and tertiary care | Multispecialty MDT meetings held once- monthly via teleconference to discuss patient cases and reach consensus. Team recommendations sent to referring care provider, added to patient’s electronic record, and discussed with patient | NR; patients not included in the actual MDT meeting, which is virtual (teleconference) | MDT meetings held once- monthly | Teleconference resources; multispecialty and multidisciplinary care providers. Total cost of funding monthly multispecialty meetings (Jan 2020–Jun 2021) was £32,400 and resulting 64 clinic appointments cost £9600 | Patients not present during the MDT/MST meeting – potential for miscommunication |  |
| Saied 2022 (UK) [congress abstract] ^25^ | NR (HF multispecialty MDT)  Type: MDT/MST meetings | MDT & MST: HF consultants and specialist nurses from primary, secondary, and tertiary care, geriatrician, nephrologist, pharmacologist, pharmacist, diabetes specialist, chest physician and palliative care physician  Program lead: NR | To utilize a multispecialty MDT to deprescribe medications as appropriate and reduce polypharmacy burden in patients with HF | A multispecialty HF MDT (lacks details) | NR | NR | Multidisciplinary care providers | NR |  |

Publications highlighted in similar colors represent the same interventions.

^a^8 countries or regions in Asia: China, Hong Kong, Malaysia, Philippines, South Korea, Taiwan, Thailand, Vietnam.
BMI, body mass index; BP, blood pressure; CAPTION, Collaboration Among Pharmacists and Physicians to Improve Outcomes Now; C.a.R.E. Clinic, Cardiac and Renal Endocrine Clinic; CareHND, Care Heart Nephrology Diabetes; CINEMA (program), Center for Integrated and Novel Approaches in Vascular-Metabolic Disease; CKD, chronic kidney disease; CMC, cardiometabolic clinic; COPD, chronic obstructive pulmonary disease; CT, computed topography; CV, cardiovascular; CVD, cardiovascular disease; DKD, diabetic kidney disease; ECG, Electrocardiogram; GP, general practitioner; HbA1c, glycated hemoglobin; HF, heart failure; HND, Heart Nephrology Diabetes (center); HTN, hypertension; ICT, information and communications technology; iCDM, Internet-based Platform for Managing Chronic Diseases; IDEALS, Integrated Diabetes Education, Awareness and Lifestyle modification in Singapore; IHD, ischemic heart disease; INSIDE (study), Integration of care for reaching targetS In Diabetic patiEnts; IT, information technology; JADE, Joint Asia Diabetes Evaluation; MDT, multidisciplinary team; MRC, Metabolic-Renal-Cardiac (clinic); MST, multispecialty team; NCT, national clinical trial; NR, not reported; PCP, primary care physician; RCT, randomized controlled trial; SBP, systolic blood pressure; T2DM, type 2 diabetes mellitus; UK, United Kingdom; USA, United States of America; V-IMPACT, Virtual Integrated Multisite Patient Aligned Care Team; wk, week.

**References**

**1.** Al-Chalabi S, Nawaz S, Alderson H, et al. An eye to the future: development of a metabolic-renal-cardiac (MRC) service. *J Am Soc Nephrol.* 2022;33.

**2.** Comaschi M, Di Lenarda A, Medea G, et al. INtegration of care for reaching targetS In Diabetic patiEnts: Design of the INSIDE Study. *Diabetes Ther.* 2020;11:359-367. doi:10.1007/s13300-019-00731-x

**3.** Chan JCN, Thewjitcharoen Y, Nguyen TK, et al. Effect of a web-based management guide on risk factors in patients with type 2 diabetes and diabetic kidney disease: a JADE randomized clinical trial. *JAMA Netw Open.* 2022;5:e223862. doi:10.1001/jamanetworkopen.2022.3862

**4.** Dubrofsky L, Lee JF, Hajimirzarahimshirazi P, et al. A unique multi- and interdisciplinary cardiology-renal-endocrine clinic: a description and assessment of outcomes. *Can J Kidney Health Dis.* 2022;9:20543581221081207. doi:10.1177/20543581221081207

**5.** Jegatheesan DK, Krishnasamy R, Stanton T, et al. Effect of a 3-year lifestyle intervention on cardiac parameters in people with CKD: sub-study of a randomized controlled trial. *J Am Soc Nephrol.* 2022;33:219.

**6.** Keel G, Muhammad R, Savage C, et al. Time-driven activity-based costing for patients with multiple chronic conditions: a mixed-method study to cost care in a multidisciplinary and integrated care delivery centre at a university-affiliated tertiary teaching hospital in Stockholm, Sweden. *BMJ Open.* 2020;10:e032573. doi:10.1136/bmjopen-2019-032573

**7.** Rafiq M, Keel G, Mazzocato P, et al. Extreme consumers of health care: patterns of care utilization in patients with multiple chronic conditions admitted to a novel integrated clinic. *J Multidiscip Healthc.* 2019;12:1075-1083. doi:10.2147/JMDH.S214770

**8.** Li H, Radhakrishnan J. A pharmacist-physician collaborative care model in chronic kidney disease. *J Clin Hypertens (Greenwich).* 2021;23:2026-2029. doi:10.1111/jch.14372

**9.** Lim LL, Lau ESH, Ozaki R, et al. Association of technologically assisted integrated care with clinical outcomes in type 2 diabetes in Hong Kong using the prospective JADE Program: a retrospective cohort analysis. *PLoS Med.* 2020;17:e1003367. doi:10.1371/journal.pmed.1003367

**10.** Narain R, Bijman L, Chen M. Progress and early outcomes of a cardiometabolic clinic in a UK tertiary cardiology centre. *Heart.* 2022;108:A96-A97.

**11.** Neeland IJ, Al-Kindi SG, Tashtish N, et al. Lessons learned from a patient-centered, team-based intervention for patients with type 2 diabetes at high cardiovascular risk: year 1 results from the CINEMA program. *J Am Heart Assoc.* 2022;11:e024482. doi:10.1161/JAHA.120.024482

**12.** Schutze A, Hohmann C, Haubitz M, Radziwill R, Benohr P. Medicines optimization for patients with chronic kidney disease in the outpatient setting: the role of the clinical pharmacist. *Int J Pharm Pract.* 2021;29:587-597. doi:10.1093/ijpp/riab033

**13.** Tan E, Khoo J, Gani LU, et al. Effect of multidisciplinary intensive targeted care in improving diabetes mellitus outcomes: a randomized controlled pilot study - the Integrated Diabetes Education, Awareness and Lifestyle modification in Singapore (IDEALS) Program. *Trials.* 2019;20:549. doi:10.1186/s13063-019-3601-3

**14.** Triantafylidis LK, Hawley CE, Fagbote C, Li J, Genovese N, Paik JM. A pilot study embedding clinical pharmacists within an interprofessional nephrology clinic for the initiation and monitoring of empagliflozin in diabetic kidney disease. *J Pharm Pract.* 2021;34:428-437. doi:10.1177/0897190019876499

**15.** Wongprasert B. IDF21-0231 Effect of multidisciplinary for improves diabetes outcomes: diabetes education, awareness and lifestyle modification. *Diabetes Res Clin Pract.* 2022;186:109502.

**16.** Anderegg MD, Gums TH, Uribe L, et al. Pharmacist intervention for blood pressure control in patients with diabetes and/or chronic kidney disease. *Pharmacotherapy.* 2018;38:309-318. doi:10.1002/phar.2083

**17.** Dixon DL, Baker WL, Buckley LF, Salgado TM, Van Tassell BW, Carter BL. Effect of a physician/pharmacist collaborative care model on time in target range for systolic blood pressure: post hoc analysis of the CAPTION trial. *Hypertension.* 2021;78:966-972. doi:10.1161/HYPERTENSIONAHA.121.17873

**18.** Lear SA, Norena M, Banner D, et al. Assessment of an interactive digital health-based self-management program to reduce hospitalizations among patients with multiple chronic diseases: a randomized clinical trial. *JAMA Netw Open.* 2021;4:e2140591. doi:10.1001/jamanetworkopen.2021.40591

**19.** Vu A, Nicholas SB, Waterman AD, et al. "Positive Kidney Health": implementation and design of a pharmacist-led intervention for patients at risk for development or progression of chronic kidney disease. *J Am Pharm Assoc (2003).* 2023;63:681-689. doi:10.1016/j.japh.2022.11.007

**20.** Burke GV, Osman KA, Lew SQ, et al. Improving specialty care access via telemedicine. *Telemed J E Health.* 2023;29:109-115. doi:10.1089/tmj.2021.0597

**21.** Katz IJ, Pirabhahar S, Williamson P, et al. iConnect CKD - virtual medical consulting: a web-based chronic kidney disease, hypertension and diabetes integrated care program. *Nephrology (Carlton).* 2018;23:646-652. doi:10.1111/nep.13070

**22.** Lu AD, Gunzburger E, Glorioso TJ, et al. Impact of longitudinal virtual primary care on diabetes quality of care. *J Gen Intern Med.* 2021;36:2585-2592. doi:10.1007/s11606-020-06547-x

**23.** Essa H, Oguguo E, Douglas H, et al. One year outcomes of heart failure multispecialty multidisciplinary team virtual meetings. *Heart.* 2021;107:A99.

**24.** Essa H, Walker L, Mohee K, et al. Multispecialty multidisciplinary input into comorbidities along with treatment optimisation in heart failure reduces hospitalisation and clinic attendance. *Open Heart.* 2022;9. doi:10.1136/openhrt-2022-001979

**25.** Saied S, Bahar J, Heron O, Mashida K, Sankaranarayanan RR. Inter-relationship of comorbidities and frailty with polypharmacy in heart failure and the role of the multispeciality MDT. *Eur J Heart Fail.* 2022;24:151.
